# Qingrehuoxue formula enhances anti-PD-1 immunotherapy in NSCLC by remodeling the tumor immune microenvironment via TREM2 signaling

**DOI:** 10.1186/s12906-025-05020-8

**Published:** 2025-07-16

**Authors:** Bin-bin Li, Yi-yang Jiang, Xue Li, Min-min Yu, Qian Meng, Dan-ni Wang, Ji-miao Zang, Fei Xu

**Affiliations:** 1 The first school of clinical medicine, Shandong University of Traditional Chinese Medicine, Jinan, 250014 China; 2 College of Acupuncture and Massage, Shandong University of Traditional Chinese Medicine, Jinan, 250014 China; 3 Department of Pathology, Affiliated Hospital of Shandong University of Traditional Chinese Medicine, Jinan, 250014 China; 4 The outpatient department, Affiliated Hospital of Shandong University of Traditional Chinese Medicine, Jinan, 250014 China; 5 Department of Respiratory and Critical Care Medicine, Affiliated Hospital of Shandong University of Traditional Chinese Medicine, Jinan, 250014 China

**Keywords:** Traditional Chinese medicine, NSCLC, TREM2, anti-PD-1, Tumor-associated macrophage

## Abstract

**Objective:**

This study evaluated the anti-cancer effect of the Qingrehuoxue Formula (QRHXF) and explored its synergistic mechanisms with anti-programmed cell death protein 1 (anti-PD-1), focusing on the tumor mircroenvironment (TME) in non-small cell lung cancer (NSCLC).

**Methods:**

The major components of QRHXF were quantified using mass spectrometry. Subcutaneous tumor mice models of Lewis lung carcinoma (LLC) were established. Mice were divided into five groups identified for pharmacodynamics: model, QRHXF (low-dose and high-dose), anti-PD-1, and anti-PD-1 + QRHXF. Tumor pathology was assessed using hematoxylin and eosin staining. Inflammatory factors were evaluated via ELISA and q-PCR. Flow cytometry was employed to quantify tumor-infiltrating immune cells. Immunofluorescence staining and western blotting (WB) were used to assess tumor angiogenesis and metastasis and confirm molecular targets and pathways.

**Results:**

Animal experiments showed that QRHXF inhibited subcutaneous tumor growth in NSCLC, with the combined therapy of QRHXF and anti-PD-1 showing superior efficacy. Particularly, QRHXF reduced extracellular matrix deposition and tumor angiogenesis to inhibit tumor metastasis. Furthermore, QRHXF downregulated tumor-infiltrating M2 macrophages and enhanced T-cell cytokine activity, upregulating the antitumor immune response. The combination of QRHXF and anti-PD-1 could augment the effects of immunotherapy. Mechanistically, QRHXF exerted its antitumor activity by inhibiting targeting triggering receptor expressed on myeloid cells 2 (TREM2) and PI3K/AKT/STAT6 pathways.

**Conclusion:**

QRHXF enhanced antitumor immune responses in NSCLC via TREM2 and modulation of the PI3K/AKT/STAT6 signaling pathway, reducing chemotactic infiltration of M2 tumor-associated macrophages within the TME. This suggests its potential as an adjuvant immune therapy for improved patient outcomes.

**Supplementary Information:**

The online version contains supplementary material available at 10.1186/s12906-025-05020-8.

## Introduction

Cancer cells evade immunosurveillance by concealing their immunogenic characteristics and fostering an environment that actively inhibits immune responses. The immune checkpoint blockade (ICB) directly modulates T cell responses by targeting immune checkpoints, thereby remodeling the tumor microenvironment (TME) [1]. Therefore, non-small cell lung cancer (NSCLC), characterized by a high antigenic load and immune infiltration, is particularly suited for ICB-based treatments [2–4]. However, TME heterogeneity contributes to variable patient responses to ICB [5]. Furthermore, tumor resistance and ICB-associated adverse effects are well recognized. Hence, identifying effective and safer therapeutic strategies is necessary to overcome these challenges in clinical practice.

Tumors recruit tumor-suppressive immune cells, such as M2 tumor-associated macrophages (TAMs), to establish an immunosuppressive TME [6]. TAMs facilitate ICB resistance by impairing antitumor immunity and promoting tumor proliferation. Particularly, they directly or indirectly suppress CD8^+^ tumor-infiltrating lymphocytes, release immunosuppressive factors such as IL-10, and enhance tumor cell growth and extravasation by fostering vascularization and extracellular matrix (ECM) remodeling [7]. Consequently, strategies to reduce the proportion of M2-TAMs and modulate their functions are promising approaches for transforming ICB-resistant tumors into ICB-sensitive ones and to impede tumor progression.

The triggering receptor expressed in myeloid cells 2 (TREM2), a plasma membrane receptor in myeloid cells, is a critical regulator in various cancers [8]. TREM2 acts principally through the DNAX-activating protein (DAP12), which transmits intracellular signals downstream via SYK and PI3K [9]. Emerging evidence indicates that TREM2 promotes M2 macrophage polarization and influences the pathogenesis of certain types of tumors by activating the PI3K/AKT pathway [10, 11] and downstream molecule STAT6 [12, 13]. Additionally, TREM2 promotes tumor growth by impairing T cell-mediated immunity and immune escape from tumors in the TME [14]. Thus, targeted therapy with anti-TREM2 antibodies can induce immune responses, inhibit tumor progression, and enhance ICB efficacy [15], making TREM2 a potential target for cancer immunotherapy.

Traditional Chinese Medicine (TCM) has emerged as an effective strategy for treating complex diseases including cancer. This is mainly attributed to its unique pharmacological characteristics, which encompass multi-component, multi-target, and multi-functional attributes. These features make TCM an appealing option for managing conditions involving complex interactions within the body [16]. Qingrehuoxue Formula (QRHXF), consisting of *Scutellaria baicalensis* and *Radix Paeoniae Rubra*, has demonstrated efficacy in managing chronic inflammatory diseases and immunocompromised conditions of the respiratory system, in addition to its antitumor effects [17–19]. We previously reported that QRHXF plays a significant role in suppressing tumor formation in vivo, primarily through the modulation of inflammation and the CXCL12/CXCR4/JAK2/STAT3 signaling pathway by regulating TAMs [17]. Notably, the P53 and GSK-3β/Nrf2 signaling pathways have been identified as key mechanisms underlying the anti-tumor effects of QRHXF [18]. Furthermore, employing a range of methodologies, including RNA sequencing, omics analysis, and experimental models such as the A549 xenograft mouse model and the subcutaneous tumor model, QRHXF has demonstrated promising potential in inhibiting the invasion and metastasis of lung cancer [19]. Subsequently, we analyzed the QRHXF components using mass spectrometry and performed individual studies on selected components. Our results demonstrated that the components, extracts, and derivatives of QRHXF possess anti-inflammatory activities and the ability to promote the remodeling of the immune microenvironment [20–22]. For instance, compounds in QRHXF, including baicalein and paeoniflorin, modulate the PI3K/AKT pathway to suppress tumor growth and metastasis by inhibiting M2 macrophage polarization. Therefore, we propose that QRHXF may suppress M2 macrophage polarization, enhance T cell-mediated innate antitumor immune response, and potentiate ICB for NSCLC treatment via TREM2 and the PI3K/AKT/STAT6 pathways.

Therefore, this study aimed to assess the antitumor properties of QRHXF and investigate its synergistic interaction with anti-programmed cell death protein 1 (anti-PD-1) therapy within the TME in mice with Lewis lung carcinoma (LLC). We believe that our results will elucidate the role of QRHXF in modulating TAMs through the TREM2 signaling pathway, offering novel directions and targets for enhancing antitumor immune responses and providing a theoretical framework for developing antitumor adjuvant drugs that enhance anti-PD-1 responses.

## Materials and methods

### Preparation of QRHXF

QRHXF was prepared according to previously established protocols [18]. The Chinese herbs, *Radix paeoniae rubra* and *Scutellaria baicalensis*, were acquired from the Affiliated Hospital of Shandong University of Traditional Chinese Medicine. Based on dose conversion by surface area, the adult (60 kg) dose of QRHXF (30 g *Scutellaria baicalensis* and 20 g *Paeonia lactiflora* per person per time) was adjusted for mice (25 g) to 0.12 g *Scutellaria baicalensis* and 0.08 g *Paeonia lactiflora* (per mouse per time).

This corresponds to daily doses of 3.79 g/kg as the low dose and 15.16 g/kg as the high dose. Dosing of the decoction was carried out at 0.2 mL per mouse per time. Aliquots were wrapped in aluminum foil, to prevent light exposure, pre-sterilized with UV light, and stored in an animal barrier facility at 4 ℃ as a backup reserve. Prior to use, the solutions were equilibrated to 37 ℃ in a water bath, mixed thoroughly, and administered orally via gavage.

### Animal experiments

Male C57BL/6J mice, four weeks old, were acquired from Jinan PengYue Laboratory Animal Co., Ltd. (China). The Animal Ethics Committee at the Affiliated Hospital of Shandong University of Traditional Chinese Medicine approved all the experimental protocols involving animals (approval number: SDSZYYAWE20240327001). At six weeks of age, each mouse was anesthetized with an intraperitoneal injection of 125 mg/kg 2,2,2-tribromoethanol (avertin) and received a subcutaneous injection of 5 × 10^5^ LLC cells in 0.1 mL of PBS into the right axillary region. Subsequently, the mice were randomly assigned to five groups: Model, QRHXF-L, QRHXF-H, Anti-PD-1, and Anti-PD-1^+^ QRHXF. The body weight and tumor volume of the mice were measured every 3 days, starting on days 3 and 6, respectively, after inoculation until the end of the experiment. Tumor volume was calculated using the following formula:

Tumor volume = length × width^2^ × 1/2.

The QRHXF-L, QRHXF-H, and QRHXF^+^ Anti-PD-1 groups received QRHXF via gavage daily, whereas the Model and Anti-PD-1 groups received equal amounts of normal saline via the same method. Mice in the Anti-PD-1 and Anti-PD-1^+^ QRHXF groups received an intraperitoneal injection of 200 µg of Anti-PD-1 (InVivoPlus anti-mouse PD-1 (CD279), BioXcell, USA) on the sixth day post-tumor inoculation. Conversely, the remaining groups were administered an equivalent volume of PBS. The treatment lasted for 21 days and all mice were sacrificed by cervical dislocation after intraperitoneal injection with 0.1% pentobarbital sodium (30 mg/kg) on day 22.

### Hematoxylin-Eosin (H&E)

Paraffin-embedded tumor tissues were cut into Sect. (5 μm thick), air-dried, deparaffinized, and stained with H&E. Thereafter, the sections were dehydrated to transparency using anhydrous ethanol and sealed using neutral glue. Images were captured using a pathological section scanner (WS-10 digitized panoramic scanner; Wisleap, Beijing, China).

### Immunohistochemistry (IHC)

After dewaxing and rehydrating the paraffin-embedded sections, antigen retrieval was performed using heat immersion in sodium citrate solution. The sections were then blocked, followed by incubation overnight at 4 °C with primary antibodies, including CD3 (1:500, 17617-1-AP, Proteintech, China), CD4 (1:500, A26036, ABclonal, China), CD8 (1:500, A23305, ABclonal, China), CD31 (1:500, A19014, ABclonal, China), CD206 (1:1000, 81525-1-RR, Proteintech, China), F480 (1:200, RT1212, HUABIO, China), LAG3 (1:200, HA721346, HUABIO, China), TIM3 (1:100, A2516, ABclonal, China), and VEGFA (1:1000, 66828-1-lg, Proteintech, China). Subsequently, the sections were then incubated with goat anti-mouse IgG (H + L) HRP (EF0001, Sparkjade, China) and goat anti-rabbit IgG (H + L) HRP (EF0002, Sparkjade, China) secondary antibodies. Color development, counterstaining, dehydration, and sealing were performed sequentially. A pathological slice scanner was used to capture images.

### Immunofluorescence (IF)

Paraffin-embedded tumor tissues were deparaffinized to facilitate antigen retrieval, followed by IF staining using primary antibodies against TREM2 (1:200, A23927, ABclonal, China), CD206 (1:200,81525-1-RR, Proteintech, China), and F4/80 (1:200, RT1212, HUABIO, China) according to the standard protocol. Subsequently, sections were incubated with goat anti-mouse IgG H + L-HRP (green, 1:200, EP0001, Sparkjade, China) and goat anti-rabbit IgG (H + L)-HRP (red, 1:200, EP0002, Sparkjade, China) secondary antibodies at 25 ℃ for 1 h. The stained sections were scanned using a digital slide scanner (Panoramic MIDI II, 3DHISTECH).

### q-PCR

All procedures were executed according to the manufacturer’s recommendations. Total RNA was isolated using the SPARKeasy Tissue/Cell RNA Rapid Extraction Kit (AC0202; Sparkjade, China). Reverse transcription was performed to obtain complementary DNA (cDNA) using the SPARKscript II All-in-one RT SuperMix for qPCR (AG0305; Sparkjade, China). The primer sequences used are presented in Supplementary Table 1. Q-PCR was conducted using the SYBR Green PCR mix (AH0104, Sparkjade, China). The cycle threshold (Ct) 2-∆∆Ct method was used to calculate the relative expression level.

### Western blot (WB)

Tumor tissue samples stored at -80 °C were separated using SDS-PAGE electrophoresis on a 10% gel and subsequently transferred onto a PVDF membrane. After incubation with a 5% skimmed milk powder blocking solution for 2 h, the membranes were exposed to primary antibodies overnight at 4 °C and then to secondary antibodies for 1 h. The primary antibodies applied were TREM2 (1:500, A23927, ABclonal, China), PI3K p110 (1:500, 20584-1-AP, Proteintech, China), p-PI3K p110 (Tyr317) (1:500, AF4369, Affinity, China), AKT (1:500, AF6261, Affinity, China), p-AKT (Ser473) (1:1000, AF0016, Affinity, China), STAT6 (1:5000, 82630-1-RR, Proteintech, China), p-STAT6 (Y641) (1:1000, AP1390, ABclonal, China), Arg-1 (1:10000, A25808, ABclonal, China), iNOS (1:2000, 80517-1-RR, Proteintech, China), MMP2 (1:1000, 10373-2-AP, Proteintech, China), MMP9 (1:1000, AB76003, Abcam, USA). The secondary antibody used was the anti-rabbit peroxidase-linked antibody (1:10000, GAR0072, MultiSciences, China). Protein bands were visualized using the ChemiDoc™ MP Imaging System. β-actin (1:10000, HRP-60008, Proteintech, China) was selected as the internal reference. ImageJ software was utilized for grayscale analysis.

### Enzyme-linked immunosorbent assay (ELISA)

The concentration of IL-6 (A20640634), IL-1β (A201B40642), TNF-α (A28240344), IFN-γ (A28040542), and TGF-β1 (A98140413) were determined in tumor tissues using an ELISA kit (Multisciences).

### Flow cytometry

Tumors derived from C57BL/6J mice were converted into single-cell suspensions using red blood cells lysis performed as per the manufacturer’s instructions. Dead cells were excluded using Zombie Dyes, and the cells were stained with anti-mouse antibodies conjugated with fluorescent markers to distinguish lymphoid and myeloid lineages. Flow cytometry data were collected using a BD Fortessa or X20 and analyzed with FlowJo Software. The reagents are detailed in Supplemental Table 2.

### Statistical analysis

The raw data are presented as the mean ± standard error of the mean (SEM). All statistical analyses were conducted using GraphPad Prism 8.0, employing a one-way analysis of variance to compare multiple sample groups. *p* < 0.05 was considered statistically significant.

## Results

### QRHXF suppressed the growth and proliferation of subcutaneous LLC tumors in vivo

**Fig. 1:**
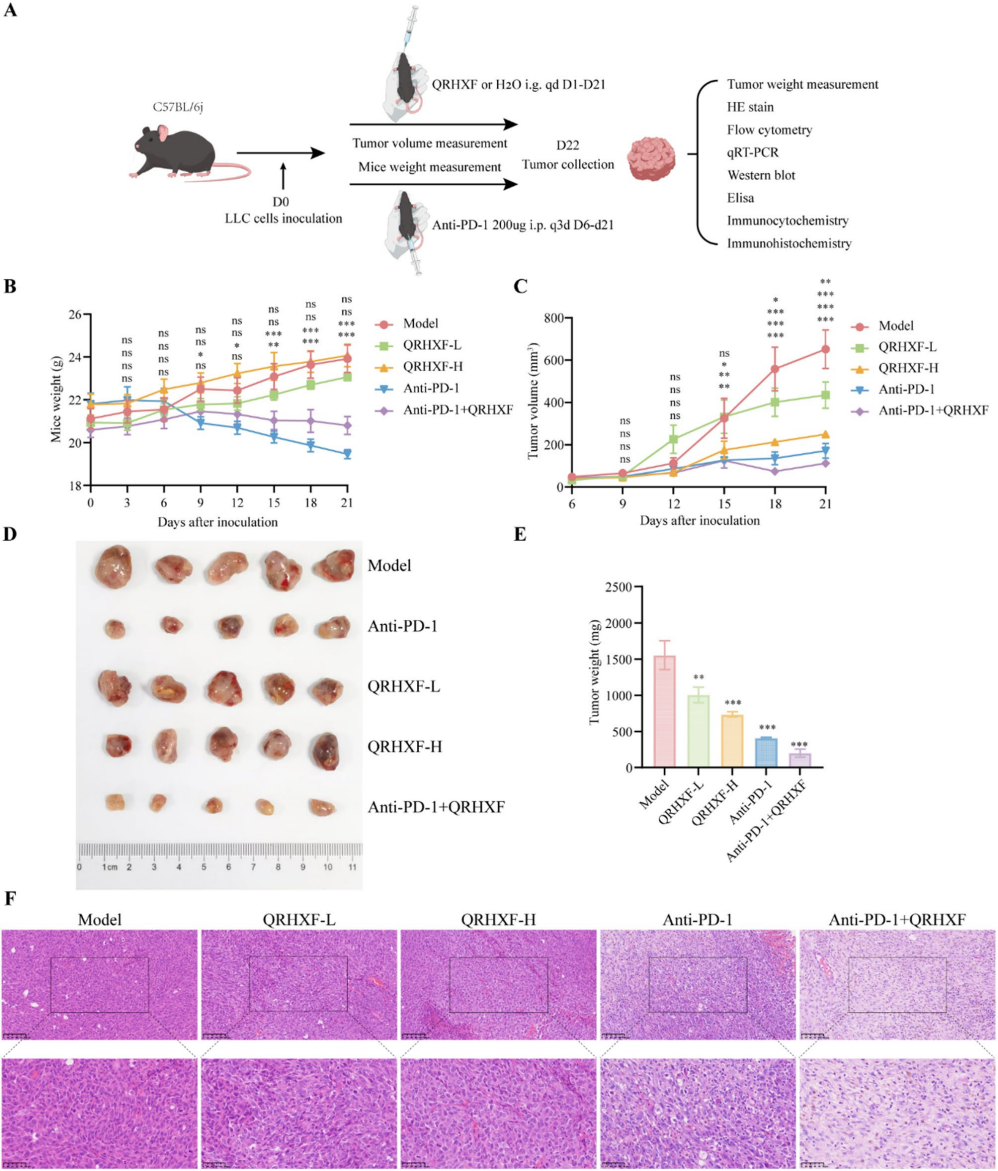
Antitumor effects of combination treatment with QRHXF and anti-PD-1 in C57BL/6 mice models bearing LLC tumor. (**A**) Flowchart of animal experimentation. (**B**) Mice body weights. (**C**) Tumor growth curve. (**D**,** E**) Tumor photographs and weight when the mice were sacrificed 21 days post-inoculation. (**F**) HE-stained images of the tumor. Values represent the mean ± SEM. *ns*: no significance, ^*^p < 0.05, ^**^p < 0.01, ^***^p < 0.001. QRHXF: Qingrehuoxue formula; LLC: Lewis lung carcinoma; HE: hematoxylin and eosin; qd: *quaque die*, q3d: *quaque 3 die*

Immunosuppressive cell populations and T cell depletion within the TME significantly impact the efficacy of immunotherapies such as anti-PD-1 treatments [1, 23, 24]. TCM prescriptions are characterized by multi-system, multi-target, and multi-directional regulation of tumor immunity. This aligns with the evolving research focus, which has shifted from directly targeting tumor cells to ameliorating the immunosuppressive conditions within the TME. In this study, we used LC-ESI-Q-TOF-MS/MS to quantitatively analyze the main components of the formula responsible for clearing heat and promoting blood circulation (Supplementary Table 3). According to the sample concentration, the content of eight active ingredients in QRHXF was calculated by the established method of HPLC-ESI-Q/TOF MS (Supplementary Table 4). Subsequently, subcutaneous LLC tumor models were constructed using C57BL/6J mice to investigate the antitumor efficacy of QRHXF and its enhanced therapeutic efficacy when combined with anti-PD-1 immunotherapy for NSCLC (Fig. 1A). As shown in Fig. 1C-E, the tumor volumes and weights of mice in all the treated groups were lower than those in the model group. The combination therapy group exhibited a significant reduction in tumor growth, attributed to synergistically enhanced therapeutic effects (*p <* 0.001). Furthermore, the relative rates of tumor proliferation (PR) and inhibition (IR) were computed, further substantiating that the combination of QRHXF and anti-PD-1 therapy effectively suppressed tumor growth (Table 1). Histopathological evaluation through HE staining revealed a notable reduction in the tumor cell density and pathological nuclear division, lighter nuclear staining, and increased necrotic areas following QRHXF and/or anti-PD-1 treatment (Fig. 1F).

**Table 1:** Relative tumor proliferation rate and Inhibition rate

Group	RTV	PR (%)	Tumor weight (mg)	IR (%)
Model	18.10 ± 5.26	100.00	1553.83 ± 198.87	0.00
QRHXF-L	15.94 ± 4.00	88.06	1006.17 ± 107.09^**^	35.25
QRHXF-H	7.75 ± 1.61	42.83	735.17 ± 36.36^***^	52.69
Anti-PD-1	5.02 ± 0.77	27.72	407.67 ± 13.06^***^	73.76
Anti-PD-1 + QRHXF	3.67 ± 0.92	20.29	200.67 ± 55.89^***^	87.09

Relative tumor volume (RTV) = tumor volume measured per measurement/tumor volume measured at day 6. Proliferation rate (PR) = RTV of treatment group/RTV of model group * 100%. Inhibition rate (IR) = 1-average tumor weight in treatment group/average tumor weight in model group * 100%. Tumor weight: Values are the mean ± SEM, *n* = 6. *ns*: no significance, ^*^p < 0.05, ^**^p < 0.01, ^***^p < 0.001. QRHXF: Qingrehuoxue formula

Body weight changes in tumor-bearing mice were systematically monitored to evaluate treatment safety and toxicity [25]. QRHXF monotherapy demonstrated favorable safety profiles, as evidenced by no statistically significant differences in mean body weight between the model group and QRHXF-treated cohorts (*p* > 0.05; Fig. 1B). In stark contrast, anti-PD-1 monotherapy induced severe weight loss, with a significant reduction in body weight compared to the model group by day 15 (*p* < 0.01). Notably, the combination of QRHXF and anti-PD-1 therapy attenuated this adverse effect: while anti-PD-1 therapy and combination groups exhibited progressive weight loss from day 9 post-modeling, the severity was attenuated in the combination therapy group. These findings collectively validated the safety of QRHXF and highlighted its capacity to mitigate anti-PD-1-associated toxicity.

### QRHXF modulated inflammatory response and reshaped the immune landscape of the TME

**Fig. 2:**
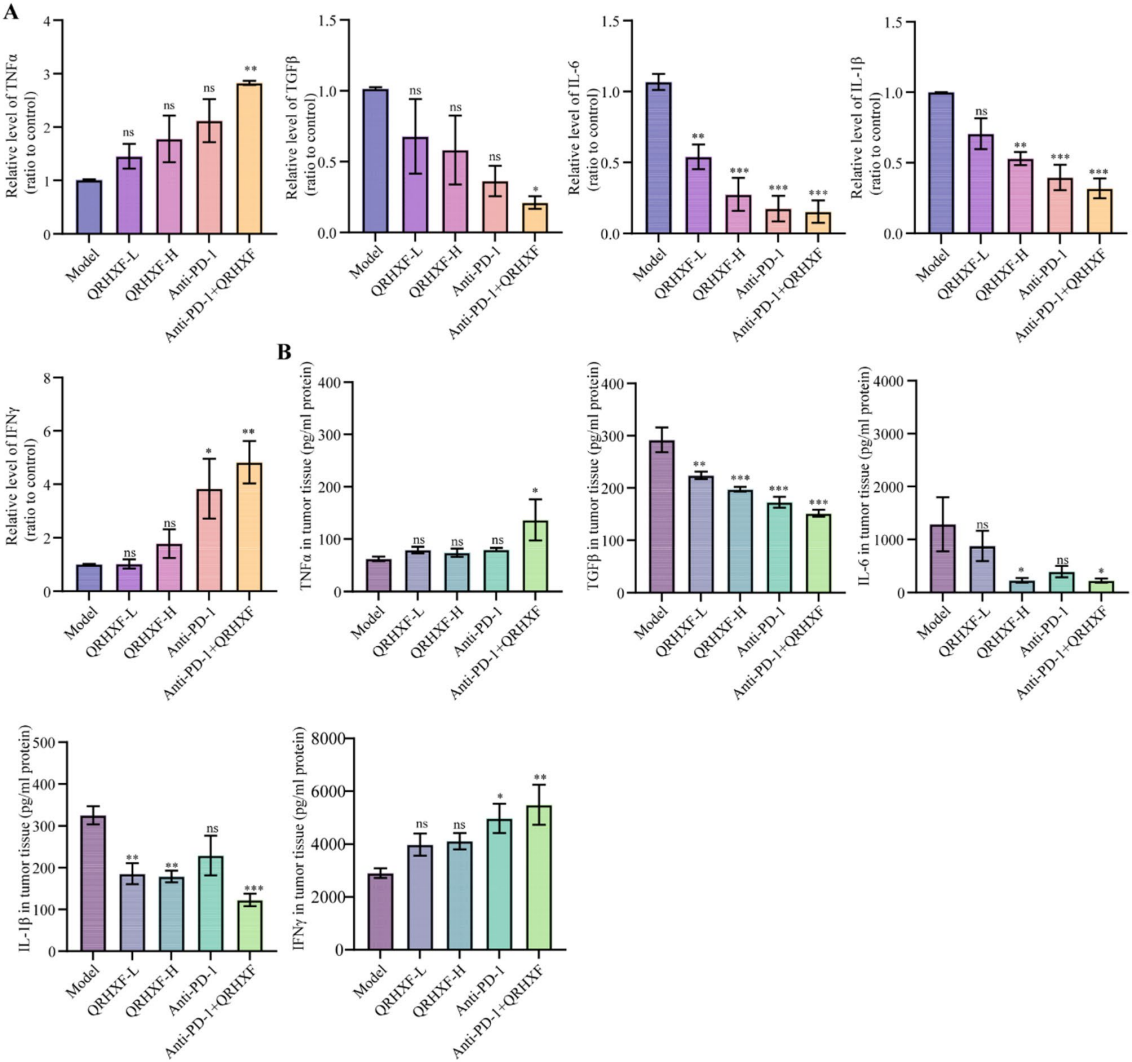
Detection of inflammation-related factors in tumors of LLC-bearing mice treated with QRHXF. (**A**) q-PCR. (**B**) ELISA. Values represent the mean ± SEM. *ns*: no significance, ^*^p < 0.05, ^**^p < 0.01, ^***^p < 0.001. ELISA: enzyme-linked immunosorbent assay. QRHXF: Qingrehuoxue formula; q-PCR: quantitative real-time polymerase chain reaction; LLC: Lewis lung carcinoma

Inflammation is a key malignant behavior associated with tumorigenesis and cancer metastasis. Chronic inflammation fosters a favorable environment for tumor development and metastasis by inducing immunosuppression [26]. Therefore, we examined the effect of QRHXF and anti-PD-1 therapies on the expression of the mRNA levels of inflammatory factor using q-PCR (Fig. 2A). IFN-γ and TNF-α, known to inhibit tumor cell proliferation, attenuate tumor-associated angiogenesis, and hinder multistage carcinogenesis [27], were significantly upregulated after treatment, particularly in the combined therapy group (*p <* 0.01). Conversely, pro-tumorigenic cytokines IL-6, IL-1β, and immunosuppressive molecules TGF-β decreased significantly after treatment (*p <* 0.05). The combination therapy group demonstrated the most significant effects. ELISA further corroborated these trends at the protein level, demonstrating that combined therapy synergistically amplified anti-inflammatory reprogramming of the TME (Fig. 2B). These findings underscore the capacity of QRHXF to counteract inflammation-driven immunosuppression.

### QRHXF suppressed NSCLC invasion and migration

**Fig. 3:**
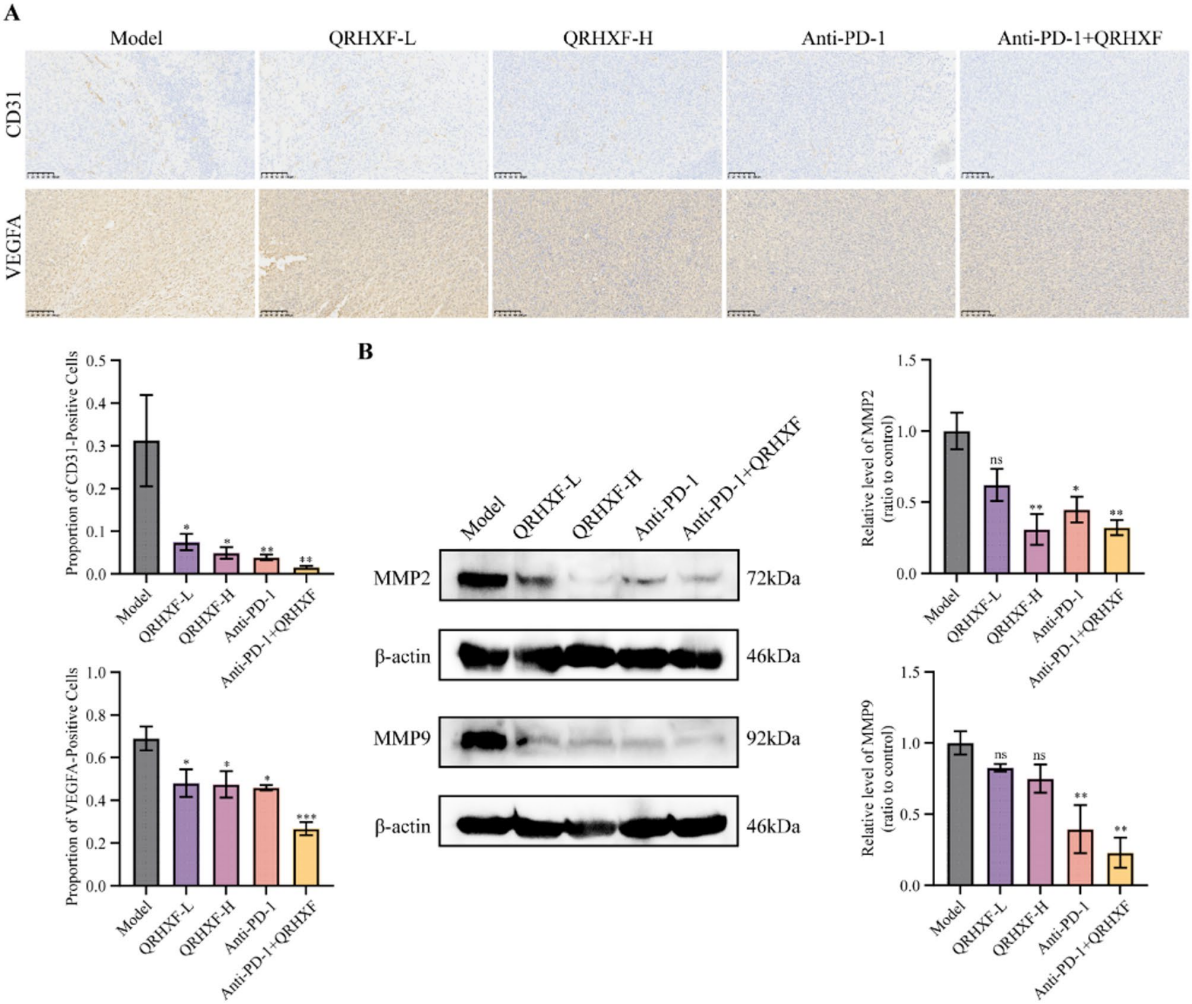
Effect of QRHXF on expression of angiogenesis-related markers. (**A**) Immunohistochemistry images showing CD31 and VEGFA expression (200× magnification). (**B**) Western blot bands and the proportions of MMP2 and MMP9. Values represent the mean ± SEM. *ns*: no significance, ^*^p < 0.05, ^**^p < 0.01, ^***^p < 0.001. QRHXF: Qingrehuoxue formula

CD31 expression levels are frequently utilized to evaluate tumor angiogenesis, confirm the presence of endothelial tissues, and assess the angiogenic potential of tumors [28]. VEGFA is a critical mediator of tumor angiogenesis [29]. Therefore, we examined the angiogenesis of the tumors using IHC (Fig. 3A). In this study, the treatment groups exhibited a significant decrease in CD31 and VEGFA expression levels relative to the model group.

Angiogenesis and ECM degradation are critical processes that facilitate the growth, migration, invasion, and metastasis of cancer cells. Matrix metalloproteinases (MMPs), particularly MMP-2 and MMP-9, are crucial enzymes that degrade the ECM, significantly contributing to the remodeling of the extracellular environment and promoting tumor progression. Our WB assay results revealed that MMP9 and MMP2 protein levels were markedly decreased in the combined therapy group (*p <* 0.01, Fig. 3B).

### QRHXF repressed the infiltration of M2-TAMs in NSCLC

**Fig. 4:**
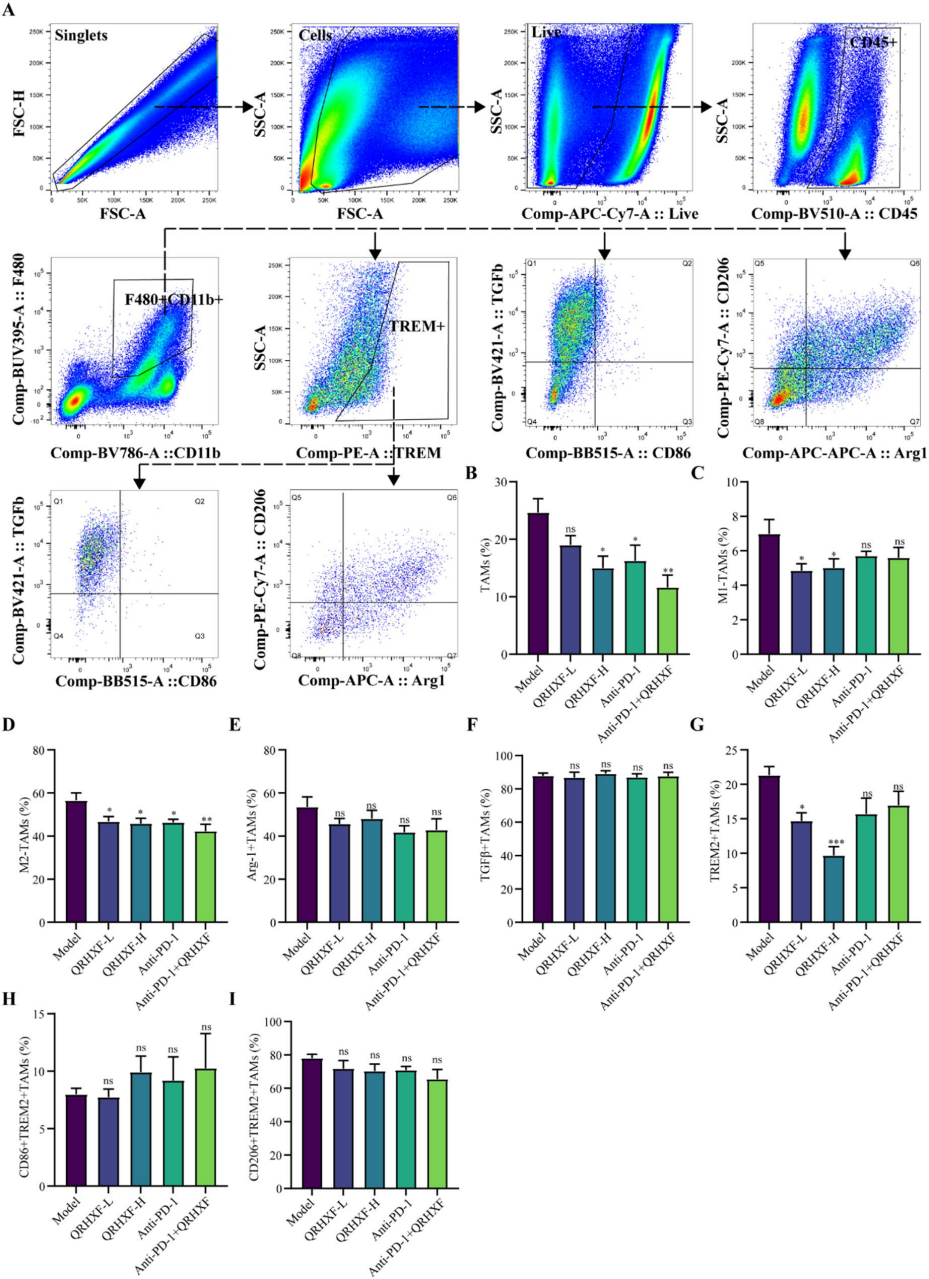
Immune cell infiltration characteristics of the macrophage-related index of QRHXF and QRHXF combined with anti-PD1 antibody. (**A**) Myeloid cell gating strategy: myeloid cells (CD45^+^CD11b^+^), TAMs (F4/80^+^), M1-TAMs (F4/80^+^CD86^+^), M2-TAMs (F4/80^+^CD206^+^). (**B-D**) Proportion of TAMs and their subtypes. (**E**,** F**) Proportion of immunosuppressive molecules TGF-β and anti-inflammatory cytokines Arg-1. (**G-I**) Proportion of TREM2, TREM2^+^M1-TAMs, and TREM2^+^M1-TAMs. Values represent the mean ± SEM. *ns*: no significance, ^*^p < 0.05, ^**^p < 0.01, ^***^p < 0.001. QRHXF: Qingrehuoxue formula

**Fig. 5:**
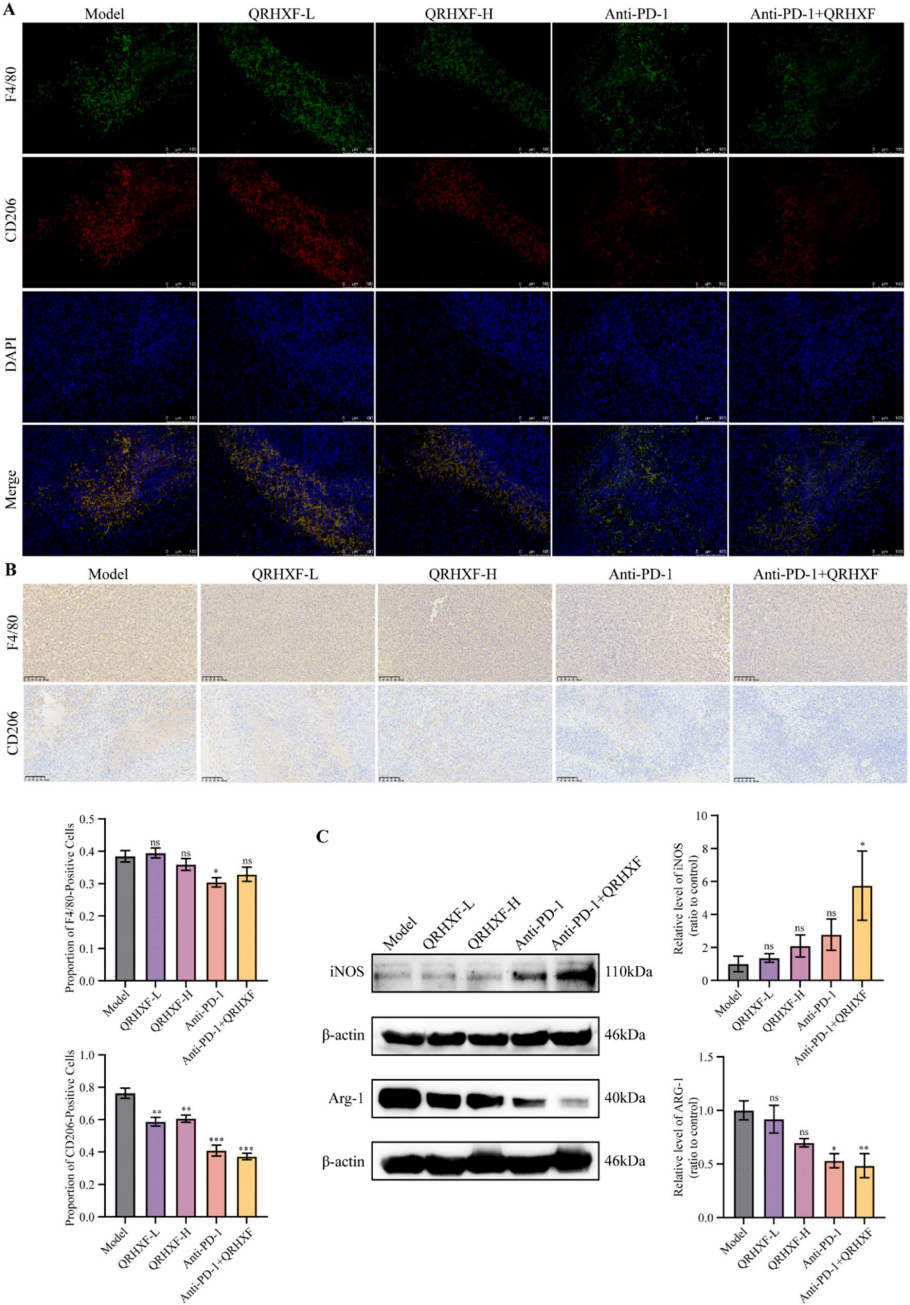
Multi-angle experimental verification of the macrophage-related index of QRHXF and QRHXF combined with anti-PD1 antibody. (**A**) Co-expression of macrophages and M2-TAMs in tumor tissues investigated using immunofluorescence. (**B**) Immunohistochemistry images for F4/80 and CD206 (200× magnification). (**C**) Comparison of the protein expression of phenotype markers associated with M1 and M2 macrophages in tumor tissues was analyzed by western blot. Values represent the mean ± SEM. *ns*: no significance, ^*^p < 0.05, ^**^p < 0.01, ^***^p < 0.001

The TME is a dynamic ecosystem comprising diverse immune cell populations, including antitumor effectors such as CD4^+^T cells, CD8^+^T cells, NK cells, dendritic cells (DCs), and M1-polarized TAMs (M1-TAMs), as well as pro-tumorigenic subsets like regulatory T cells, M2-TAMs, and myeloid-derived suppressor cells. Among these, M2-TAMs are pivotal contributors to tumor progression, immune evasion, and therapeutic resistance by fostering an immunosuppressive milieu [30]. Therefore, we systematically evaluated immune cell infiltration patterns to investigate the impact of QRHXF on TME remodeling (Fig. 4A). Flow cytometry analysis of the myeloid compartment (CD45^+^CD11b^+^cells) revealed that QRHXF monotherapy significantly reduced the proportion of M2-like TAMs (F4/80^+^CD206^+^) compared to the model group (*p <* 0.05; Fig. 4B-D). Notably, the combination of QRHXF and anti-PD-1 therapy exhibited a more pronounced reduction in M2-TAMs (*p* < 0.01), underscoring the synergistic immunomodulatory effects. Subsequently, we employed IF (Fig. 5A) and IHC (Fig. 5B) to visualize macrophage infiltration and corroborate these findings. The results demonstrated that the alterations observed following pharmacological intervention in M2-TAMs were consistent with the flow cytometry findings. Notably, the expression of Arg-1—a hallmark of M2-TAMs linked to immunosuppression and tumor progression [31]—showed a downward trend in Arg-1^+^TAMs post-treatment, although statistical significance was not reached (*p* > 0.05; Fig. 4E). However, WB revealed the elevated expression of the M1 marker iNOS (*p* < 0.05) alongside reduced levels of the M2 marker Arg-1 (*p* < 0.01) (Fig. 5C). This discrepancy may stem from methodological differences, as WB quantifies total protein levels, whereas flow cytometry assesses cellular subpopulations, potentially influenced by spatial heterogeneity within the tumor. While flow cytometric analysis did not reveal statistically significant alterations in Arg-1 expression, QRHXF or/and anti-PD-1 treatment reduced the recruitment of CD206^+^TREM2^+^TAMs, implicating TREM2 as a potential mediator of the immunomodulatory activity by QRHXF. Collectively, these results indicated that QRHXF suppressed M2-associated pro-tumorigenic functions.

Extensive studies have established that TGF-β^+^TAMs drive tumor progression by inducing EMT and remodeling the immunosuppressive TME [32]. Although QRHXF treatment significantly reduced M2-polarized macrophage infiltration (*p* < 0.05), the proportion of TGF-β^+^TAMs remained unchanged across experimental groups (Fig. 4F, *p* > 0.05). This apparent dissociation between M2 reduction and stable TGF-β^+^TAM levels may reflect distinct regulatory mechanisms. Thus, QRHXF could selectively modulate M2 polarization markers (e.g., CD206/Arg1) without directly targeting TGF-β production or storage in TAMs. Alternatively, TGF-β signaling might be sustained through compensatory pathways, such as protease-mediated activation of latent TGF-β pools in the TME [33] or TGF-β secretion by non-macrophage stromal cells, thereby maintaining pathway activity despite shifts in macrophage phenotypes.

Additionally, TREM2, a determinant of the TAM phenotype in the TME [34], was enriched in tumors and associated with inhibitory TAMs [35]. Figure 4G illustrated that QRHXF significantly reduced the proportion of TREM2^+^TAMs within the CD11b^+^ myeloid population (*p* < 0.05), a phenomenon not observed in the anti-PD-1 monotherapy groups. Therefore, we conducted a detailed analysis of the proportions of M1-TAMs and M2-TAMs in the TREM2^+^TAMs cell population (Fig. 4H, I). Although not statistically significant, QRHXF or/and anti-PD-1 treatment reduced the recruitment of CD206^+^TREM2^+^TAMs, implicating TREM2 as a potential mediator of the immunomodulatory activity by QRHXF.

### QRHXF alleviated T cell exhaustion and augmented CD8^+^T cell activation in NSCLC

**Fig. 6:**
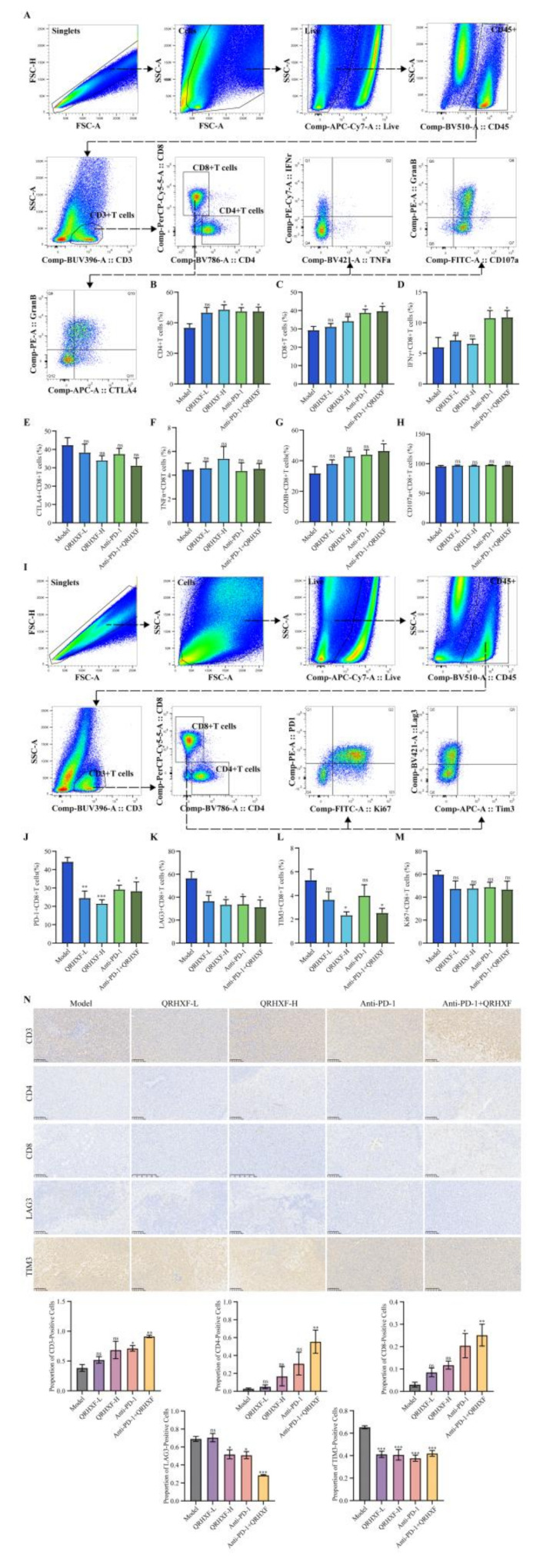
Immune cell infiltration characteristics of the lymphocyte-related index of QRHXF and QRHXF combined with anti-PD1 antibody. (**A**,** I**) Lymphocyte gating strategy: T cells (CD45^+^CD3^+^), CD4^+^T cells (CD4^+^CD8^−^), CD8^+^T cells (CD4^−^CD8^+^). (**B**,** C**) Proportion of T cells and their subtype. (**D**,** F**,** H**) Comparison of the expression of effector molecules (CD107a, IFN-γ, TNF-α) by CD8^+^T cells. (**E**,** J-L**) Comparison of the expression of coinhibitory receptors (CTLA4, PD-1, LAG3, TIM3) on CD8^+^T cells. (**G**) Comparison of the expression of GZMB on CD8^+^T cells. (**M**) Comparisons of the expression of Ki-67 on CD8^+^T cells. (**N**) Immunohistochemistry images for CD3, CD4, CD8, LAG3, and TIM3 (200×). Values represent the mean ± SEM. *ns*: no significance, *p < 0.05, **p < 0.01, ***p < 0.001. QRHXF: Qingrehuoxue formula

In an established TME, infiltrated T cells frequently exhibit functional exhaustion. Macrophages contribute critically to immune evasion by restricting CD8^+^T cell migration into tumor islets, effectively excluding these cytotoxic lymphocytes from the tumor core [36]. This macrophage-mediated suppression of T cell trafficking represents a key mechanism of tumor immune escape. Therefore, we evaluated the effect of QRHXF and its combination with anti-PD-1 therapy on the T cell effector function (Fig. 6A, I).

The proportions of CD4^+^and CD8^+^T cells in the CD45^+^CD3^+^T cell population were significantly higher in the treatment groups compared to the model group (*p <* 0.05; Fig. 6B, C). CD8^+^T cells, which secrete TNF-α and IFN-γ are essential effectors involved in tumor cell elimination, whereas CD4^+^T cells are pivotal in orchestrating the overall antitumor response [37, 38]. However, under the effect of multifaceted inhibitory signals, exhausted CD8^+^T cells express inhibitory receptors such as LAG-3, PD-1, CTLA-4, and TIM-3 [39]. Therefore, we assessed the effects of QRHXF on the CD8^+^T cell functionality by analyzing the effector molecule and inhibitory receptor expression on T cells in NSCLC. Functional analysis revealed dual effects on CD8^+^T cells, demonstrating that the treatment group exhibited increased proportions of IFN-γ^+^CD8^+^T cells (Fig. 6D)—critical for tumor cell cytotoxicity—alongside reduced expression of CTLA-4, PD-1, LAG-3, and TIM-3 on CD8^+^T cells (Fig. 6E, J-L) compared to the model group, collectively suggesting restored immune cell activity. Moreover, the percentage of TNF-α^+^CD8^+^T cells exhibited only a non-significant upward trend in the monotherapy groups with no observable change in the combination therapy group (Fig. 6F).

GZMB, a serine protease located in the granules of cytotoxic lymphocytes, alongside perforin, is responsible for reflecting the activity of antitumor immune responses [40]. CD107a expression correlates closely with the activity of NK cells or T cells [41]. We found that combination therapy boosted cytotoxic potential through increased GZMB^+^CD8^+^T cells (*p <* 0.05; Fig. 6G), although CD107a^+^CD8^+^T cells (a degranulation marker) remained unchanged (*p* > 0.05, Fig. 6H), suggesting selective enhancement of lytic machinery over degranulation capacity. Notably, despite the overall expansion of tumor-infiltrating T cells, Ki67^+^CD8^+^T cell proportions trended downward non-significantly across groups, implying that the augmented infiltration likely stemmed from enhanced recruitment or survival rather than in situ proliferation (Fig. 6M). This dissociation between T cell influx and proliferative activity underscores the capacity of QRHXF to restore effector function in pre-existing CD8^+^T cells while countering exhaustion-driven immune suppression. Immunohistochemical staining further confirmed these dynamics (Fig. 6N), with expanded CD3/CD4/CD8 populations and diminished LAG3/TIM3 cells collectively illustrating the synergy of QRHXF with anti-PD-1 therapy in remodeling the immunosuppressive TME through coordinated modulation of T cell recruitment, functional reprogramming, and exhaustion reversal.

These findings indicated that QRHXF and its combination with anti-PD-1 therapy significantly enhanced CD4^+^and CD8^+^T cell infiltration in LLC-bearing mouse tumors, suggesting that QRHXF could significantly influence the regulation of TME. Moreover, QRHXF mitigated the depletion of CD8^+^T cells and restored CD8^+^T cell activation, potentially enhancing the efficacy of anti-PD-1 and anti-tumor immune responses.

### QRHXF suppressed the expression of TREM2 and the PI3K/AKT/STAT6 pathway in NSCLC

**Fig. 7:**
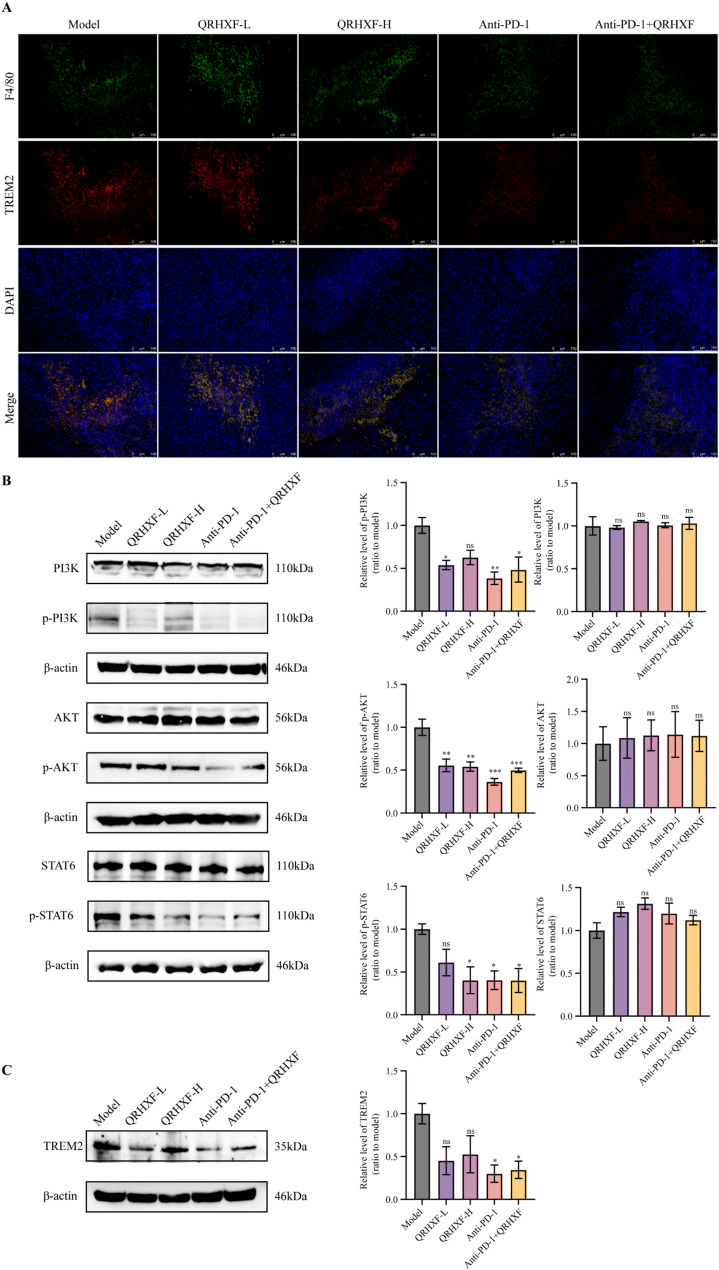
Effect of QRHXF on the TREM2 and PI3K/AKT/STAT6 pathway and investigations into its upstream targets. (**A**) Co-expression of macrophages and TREM2 in tumor tissues investigated using immunofluorescence. (**B**,** C**) Comparison of TREM2 and PI3K/AKT/STAT6 pathway-related protein expression in tumor tissues analyzed by western blot. Values represent the mean ± SEM. *ns*: no significance, ^*^p < 0.05, ^**^p < 0.01, ^***^p < 0.001. QRHXF: Qingrehuoxue formula

We performed IF and WB assays on key proteins of the PI3K/AKT/STAT6 pathways to elucidate the molecular mechanism by which QRHXF promotes metastasis in tumor cells and augments checkpoint immunotherapy. IF staining confirmed the binding of macrophages to the TREM2 cell surface, identifying the cellular localization of macrophages and TREM2. Additionally, IF staining revealed that TREM2 colocalized exclusively with F4/80 positive cells (Fig. 7A). The WB results showed a decrease in the ratio of phosphorylated PI3K (p-PI3K Tyr317), AKT (p-AKT S473), and STAT6 (p-STAT6 Y641) compared to the model group (Fig. 7B), along with reduced TREM2 protein levels (Fig. 7C). Thus, QRHXF suppressed TREM2 expression and the PI3K/AKT/STAT6 pathway activation.

## Discussion

Tumor resistance may stem from an interaction between tumor heterogeneity and an immunosuppressive TME, which limit the efficacy of immunotherapies. TAMs, which are crucial constituents of the TME, play a significant role in cancer progression and metastasis. Macrophages exhibiting an M2-like phenotype enhance the expression of immunosuppressive surface proteins and anti-inflammatory factors, thereby impairing the activation and function of effector T cells and further strengthening the immunosuppressive nature of the microenvironment. They secrete cytokines that promote T-cell depletion while simultaneously reducing the capacity of CD8^+^and CD4^+^T cells to eradicate tumor cells within the TME, contributing to tumor progression, immune evasion of neoplastic cells, and resistance to cancer immunotherapy.

**Fig. 8:**
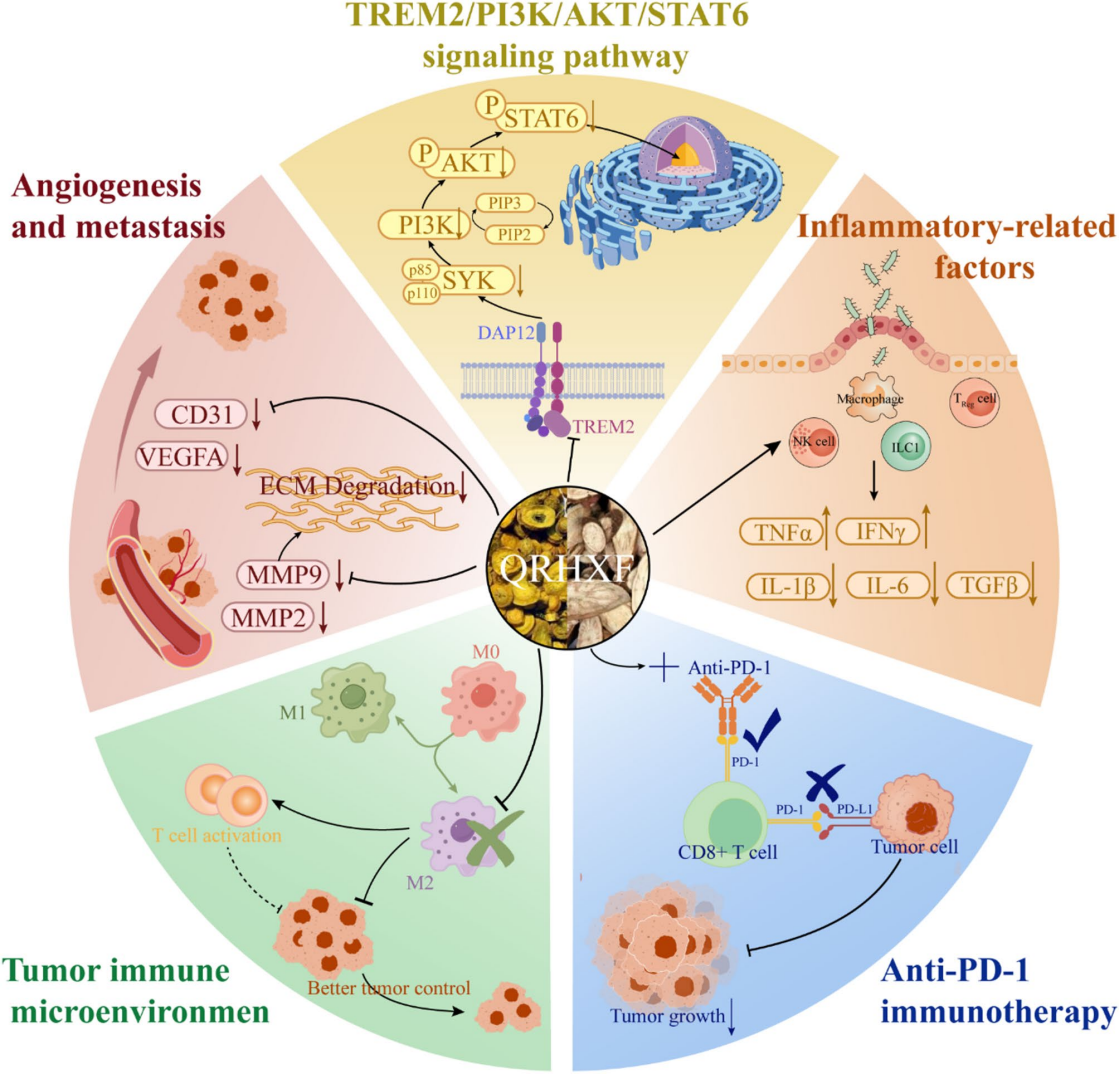
Antitumor mechanism diagram of QRHXF QRHXF: Qingrehuoxue formula

We previously showed that QRHXF is a classic TCM prescription, which activates anti-inflammatory and tolerance mechanisms, remodels the immune microenvironment, and inhibits antitumor responses [18, 19]. The main active components of QRHXF, such as baicalin, baicalein, and paeoniflorin, identified by mass spectrometry, have been shown to modulate the TME and inhibit tumor growth and metastasis [42–44]. Based on these insights, we investigated the anti-NSCLC efficacy of QRHXF and its mechanism of inducing immune microenvironment remodeling and potentiating immunotherapies in terms of tumor immunity (Fig. 8).

We evaluated the efficacy of QRHXF alone and in combination with anti-PD-1 therapy. The results revealed that while QRHXF significantly inhibited tumor growth and proliferation, its combination with anti-PD-1 therapy achieved superior efficacy than monotherapy for anti-NSCLC. Body weight fluctuations in experimental animals, commonly used as an index to assess the overall health condition in vivo studies [45], revealed a notable enhancement in the quality of life for mice that were treated with QRHXF as an adjuvant therapy.

The TME is modulated by various cytokines that orchestrate a diverse range of biological functions, including immunomodulation, suppression of ongoing inflammation, and promotion of tissue repair and regeneration. TNF-α exhibits anticancer properties by inducing necrosis in tumor tissues [7]. Specifically, TNF-α plays a specific role in regulating tumor development, invasion, metastasis, acquired resistance, adaptive immune, and innate immune. However, its accumulation in the TME has been implicated in chemoresistance in certain cancers. IFN-γ, primarily secreted by activated T cells and NK cells upon viral, mitogenic, and double-stranded RNA stimulation, plays a vital role in anti-viral and antitumor immunity. TGF-β, derived from tumor cells, adjacent stromal cells, and tumor-infiltrating inflammatory cells, contribute to tumor cell growth, invasion, and spread [46]. Additionally, TGF-β signaling axes have been recognized as an EMT-inducing factor in hepatocellular carcinoma [47]. IL-6 is a pleiotropic cytokine that promotes inflammation and tumor cell proliferation, enhancing tumor cell migration, invasion, and metastasis. Similarly, persistent overactivation of the IL-1β pathway has been linked to tumor promotion, highlighting the therapeutic potential of IL-1β inhibition in cancer prevention and treatment [48, 49]. Therefore, we evaluated the impact of QRHXF and its combination with anti-PD-1 therapy on inflammatory factors in NSCLC. TNF-α and IFN-γ were significantly upregulated, while TGF-β, IL-6, and IL-1β were notably downregulated following treatment. These findings indicated that QRHXF could modulate signaling pathways to mitigate tumor resistance.

Furthermore, tumor angiogenesis and ECM depletion are significantly influenced by driving tumor growth, invasion, and migration. Pro-inflammatory M1 macrophages release inflammatory mediators such as IL-6, TNF-α, and iNOS, inciting robust pro-inflammatory immune responses that inhibit tumor cell proliferation [50–53]. However, M2-polarized macrophages also highly express Arg-1, which are consumed during proline and polyamine biosynthesis [54]. M2-TAMs release various proteolytic enzymes, including MMP2 and MMP9, which facilitate ECM breakdown and tumor progression. Additionally, M2-TAMs produce ECM components such as osteopontin and fibronectin, which stabilize the tumor stroma and promote cell adhesion. Moreover, tumor cells produce IL-1β, CCL, VEGF, and SDF-1α, which recruit macrophages to the TME, facilitating inflammation, angiogenesis, and tumor growth [7]. CD31, a canonical marker of tumor vasculature, is implicated in cell migration and invasion in advanced-stage tumors. We found that QRHXF reduced the number of CD31 and VEGFA positive cells, suppressing tumor growth by inhibiting angiogenesis. QRHXF also decreased MMP2 and MMP9 protein expression levels, mitigating ECM degradation in NSCLC and suppressing tumor migration and invasion. The WB assay showed that QRHXF significantly inhibited the TME-induced upregulation of Arg-1 expression and downregulation of iNOS expression. These findings confirmed that QRHXF suppressed NSCLC cell growth and metastasis by modulating TME, angiogenesis, and ECM remodeling. Moreover, QRHXF enhanced the antitumor effect of anti-PD-1 therapy against NSCLC.

Subsequently, we performed flow cytometry assays on immune cell populations to investigate the potential impact of QRHXF on the immune microenvironment. QRHXF exhibited the potential to promote antitumor immunity by regulating the immune system. Within the population of bone marrow cells, QRHXF reduced the proportion of M2-type macrophage polarization, compared to the model group.

Additionally, we investigated the density and tissue distribution of TREM2^+^TAMs using flow cytometry because TREM2 fosters an immunosuppressive TME via TAMs. The population of CD206^+^TREM2^+^TAMs had an increased trend in the treatment groups, with a better effect observed in the Anti-PD-1 + QRHXF group, indicating that QRHXF may function as an oncogene by targeting TREM2 to regulate the TME and improve the efficacy of immunosuppressives.

Subsequently, we examined the changes in the lymphoid subgroups using flow cell sorting before and after treatment and found that CD8^+^ and CD4^+^T cells were significantly increased, suggesting that these cells play critical roles in tumor cell eradication. T cell exhaustion is characterized by elevated expression of inhibitory receptors including PD-1, LAG3, and TIM3, while markers such as Ki67 serve as indicators of T cell activation and proliferative capacity. Notably, CD8 + T cells execute antitumor functions through dual mechanisms: immunomodulation via effector cytokines like TNF-α and IFN-γ and direct cytotoxicity mediated by granzyme/perforin release—biomolecules also associated with CD107a expression that collectively drive antitumor immunity. Paradoxically, within the TME, persistent antigen exposure drives CD8 + T cell functional exhaustion, progressively impairing their cytotoxic potential and cytokine production. Our results demonstrated that combining QRHXF with anti-PD-1 therapy reversed the depletion of CD8^+^T cells and downregulated chemokine ligands and receptors, thereby alleviating TME-associated immunosuppression in NSCLC and enhancing the efficacy of immunotherapy.

Thereafter, we investigated the TREM2/PI3K/AKT/STAT6 pathway to delve into the mechanisms that possibly underlie the effects of QRHXF on the TME. TREM2, a receptor critical for regulating adaptive and innate immunity [55], has been implicated in promoting immunosuppression within the TME [56]. Conversely, TREM2 deficiency has been shown to mediate TME remodeling, significantly enhancing tumor resistance in mice compared to wild-type counterparts [15]. Thus, high levels of TREM2 in M2 macrophages can serve as a biomarker for isolating these cells from macrophages [57, 58]. Additionally, TREM2^+^TAMs have been identified in the TME of multiple tumor types and are associated with patients who exhibit resistance or non-responsiveness to ICB treatment [59]. Consequently, inhibiting TREM2 specifically changes the phenotype and function of TAMs, thereby improving the immunotherapy effectiveness. The TREM2 receptor also plays a role in suppressing T cell activation [15, 34]. A study reported that TREM2^+^TAMs influenced the immunosuppressive activity of regulatory T cells in NSCLC and enhanced CD8^+^ T cell exhaustion [56]. Furthermore, TREM2^+^DCs significantly suppress T cell proliferation in lung cancer [60]. In contrast, TREM2 knockdown enhances peripheral immune cell infiltration and activation, laying a foundation for ICB-based therapies in tumors with immunosuppressive TME or poor T cell infiltration [61].

Furthermore, numerous studies have reported that TREM2 acts as an oncogene, advancing cancer by triggering the PI3K/AKT and JAK/STAT3 pathways [14]. STAT6 is a crucial regulatory transcription factor for M2 macrophage polarization [62]. Although JAK1 is the primary upstream activator of STAT6, other intracellular activation molecules of serine kinases such as AKT and mTOR, also promote STAT6 dimerization and phosphorylation [63–65]. The phosphorylated STAT6 translocates to the nucleus, activating the transcription of M2 phenotype related genes [66]. Additionally, a recent study revealed that the PI3K/AKT/mTORC2 pathway activation, along with STAT6 downstream signaling, contributes to lipid metabolism imbalances within macrophages related to IL-4-induced M2 polarization [65]. Similarly, LMP7/alveolar macrophages promote M2 macrophage polarization by activation of the PI3K/AKT/STAT6 signaling pathway while also enhancing the expression of IRF4 [67]. Moreover, the main components of QRHXF, such as paeoniflorin [44] and baicalein [68] inhibit cell viability and induce apoptosis by suppressing the PI3K/AKT signaling pathway in several cancers.

Thus, we explored whether QRHXF could regulate macrophage polarization, enhance T cell response, and potentiate antitumor efficacy by remodeling the TME via the TREM2/PI3K/AKT/STAT6 signaling pathway. Our results demonstrated that QRHXF could reduce TREM2 expression levels and inhibit the activation of these pathways triggered by phosphorylation, highlighting its potential as an antitumor therapeutic. The observed antitumor effects likely result from the combined effects of the various components of QRHXF. Meanwhile, anti-PD-1 treatment enhanced the infiltration of activated T cells, and its combination with QRHXF synergistically inhibited tumor growth and improved survival rates.

Nonetheless, this study has some limitations that warrant further investigation. First, although the data suggest that QRHXF remodels the TME via TREM2 signaling, the causal role of TREM2 remains unvalidated, because genetic approaches (e.g., TREM2 knockdown or overexpression) were not employed. Second, this study focused on the PI3K/AKT/STAT6 pathway, leaving other potential regulatory axes (e.g., JAK/STAT3, NF-κB) unexplored, necessitating systematic multi-omics analyses to fully delineate the polypharmacological effects of QRHXF. Third, as a compound formula, the individual contributions of individual active components in QRHXF (e.g., baicalein, paeoniflorin) to immune modulation remain unclear, requiring component-specific pharmacodynamic studies. Finally, the mechanistic interplay between TREM2 and TAM functional states—such as lipid metabolism reprogramming or intercellular crosstalk—merits further exploration to refine the proposed pathway-centric framework. Consequently, addressing these gaps will advance the rational design of QRHXF-based combinatorial regimens for NSCLC immunotherapy.

## Conclusion

This study demonstrated that QRHXF potentiates antitumor immune responses by inhibiting TAM polarization into the M2 phenotype and enhancing CD8^+^T cell recruitment and activation via the TREM2 signaling pathway. Additionally, the combination of QRHXF and anti-PD-1 therapy exhibited significant synergistic antitumor effects in vivo, demonstrating its potential as an innovative cancer immunotherapy strategy. Thus, our study provides compelling evidence for the antitumor potential of QRHXF against NSCLC, offering a promising direction for adjuvant immune therapy. Therefore, further research is necessary to replicate and expand upon these findings.

## Electronic supplementary material

Below is the link to the electronic supplementary material.

Supplementary Material 1

Supplementary Material 2

Supplementary Material 3

Supplementary Material 4

Supplementary Material 5

## Data Availability

The datasets used and/or analysed during the current study are available from the corresponding author on reasonable request.
