# Supplementary material for: Qingrehuoxue formula enhances anti-PD-1 immunotherapy in NSCLC by remodeling the tumor immune microenvironment via TREM2 signaling
Source: BMC Complement Med Ther. 2025 Jul 16;25:270. doi: 10.1186/s12906-025-05020-8 (PMC12269164; doi:10.1186/s12906-025-05020-8)
Supplement: Supplementary file 1 — Supplementary Material 1 [file 12906_2025_5020_MOESM1_ESM.docx]

**Supplementary table 1.** **Primer sequences and PCR conditions for the investigated genes.**

| Gene |  | Sequence (5' to 3') |
| --- | --- | --- |
| IL-6 | forward | GAG AGG AGA CTT CAC AGA GGA TAC C |
|  | reverse | TCA TTT CCA CGA TTT CCC AGA GAA C |
| TNF-α | forward | ACG TGG AAC TGG CAG AAG AGG |
|  | reverse | TGA GAA GAG GCT GAG ACA TAG GC |
| IL-1β | forward | TCG CAG CAG CAC ATC AAC AAG |
|  | reverse | TCC ACG GGA AAG ACA CAG GTA G |
| TGF-β | forward | ACC GCA ACA ACG CCA TCT ATG AG |
|  | reverse | GGC ACT GCT TCC CGA ATG TCT G |
| IFN-γ | forward | CTG GAG GAA CTG GCA AAA GGA TGG |
|  | reverse | GAC GCT TAT GTT GTT GCT GAT GGC |
| β-actin | forward | AGC CAT GTA CGT AGC CAT CC |
|  | reverse | CTC TCA GCT GTG GTG GTG AA |
