# Supplementary material for: Qingrehuoxue formula enhances anti-PD-1 immunotherapy in NSCLC by remodeling the tumor immune microenvironment via TREM2 signaling
Source: BMC Complement Med Ther. 2025 Jul 16;25:270. doi: 10.1186/s12906-025-05020-8 (PMC12269164; doi:10.1186/s12906-025-05020-8)
Supplement: Supplementary file 2 — Supplementary Material 2 [file 12906_2025_5020_MOESM2_ESM.docx]

**Supplementary table 2.** **Antibodies and reagents utilized for flow cytometry analysis.**

| **Reagent name** | **Catalog numbers** | **Company names** | **Dilution ratio** |
| --- | --- | --- | --- |
| Collagenase IV | C5138 | Sigma |  |
| DNase I | D4527 | Sigma |  |
| Anti-mouse CD16/32 block | 14-0161-86 | Thermo | 1:100 |
| Zombie fixable live dead viability dye APC-Cy7 | 65-0865-18 | Thermo | 1: 200 |
| Mouse CD45-BV510 antibody | 103138 | BioLegend | 1: 100 |
| Mouse CD11b-BV786 antibody | 417-0112-82 | Thermo | 1:100 |
| Mouse F480 BUV395 antibody | 565614 | BD | 1:100 |
| Mouse CD86 FITC antibody | 11-0862-85 | Thermo | 1:100 |
| Mouse CD206 PE-Cy7 antibody | 141720 | BioLegend | 1: 100 |
| Mouse TREM2-PE antibody | FAB17291P | RD | 1: 100 |
| Mouse Arg1 APC antibody | 17-3697-82 | Thermo | 1:100 |
| Mouse TGFb-BV421 antibody | 141408 | BioLegend | 1: 100 |
| Mouse CD3 BUV395 antibody | 563565 | BD | 1:100 |
| Mouse CD4-BV786 antibody | 417-0042-82 | Thermo | 1:100 |
| Mouse CD8 PerCP-Cy5.5 antibody | 100734 | BioLegend | 1: 100 |
| Mouse IFNr PE-Cy7 antibody | 505826 | BioLegend | 1: 100 |
| Mouse TNFa-BV421 antibody | 506327 | BioLegend | 1: 100 |
| Mouse CTLA4 APC antibody | 17-1522-82 | Thermo | 1:100 |
| Mouse GranB PE antibody | 396406 | BioLegend | 1: 100 |
| Mouse CD107a FITC antibody | 53-1071-82 | Thermo | 1:200 |
| Mouse CD8 PE-Cy7 antibody | 25-0081-82 | Thermo | 1:200 |
| Mouse Ki67 FITC antibody | 11-5698-80 | Thermo | 1:200 |
| Mouse PD1-PE antibody | 551892 | BD | 1:100 |
| Mouse Lag3-BV421 antibody | 740072 | BD | 1:100 |
| Mouse Tim3-APC antibody | 134008 | BioLegend | 1: 100 |
| Intracellular Fix/Perm Buffer Set | 88-8824-00 | Thermo |  |
| Foxp3 Transcription Factor Staining Buffer Set | 00-5523-00 | Thermo |  |
| Leukocyte Activation Cocktail, with BD GolgiPlug | 550583 | BD | 1: 500 |
