# Supplementary material for: Qingrehuoxue formula enhances anti-PD-1 immunotherapy in NSCLC by remodeling the tumor immune microenvironment via TREM2 signaling
Source: BMC Complement Med Ther. 2025 Jul 16;25:270. doi: 10.1186/s12906-025-05020-8 (PMC12269164; doi:10.1186/s12906-025-05020-8)
Supplement: Supplementary file 3 — Supplementary Material 3 [file 12906_2025_5020_MOESM3_ESM.docx]

**Supplementary table 3.** **Standard curves, detection ions and detection ranges of 8 compounds in QRHXF.**

| No. | Compound Name | | t_R_ (min) | MS (m/z) | Regression Equation | r^2^ | Test Range (ug/mL) |
| --- | --- | --- | --- | --- | --- | --- | --- |
| 1 | | Albiflorin | 4.67 | 525.1608  [M+COOH-H]^-^ | Y=152779*X-3661 | 0.9993 | 0.132-8.48 |
| 2 | | Paeoniflorin | 5.13 | 525.1608  [M+COOH-H]^-^ | Y=179016*X-15742 | 0.9933 | 0.312-19.9 |
| 3 | | Baicalin | 7.25 | 445.0771 [M-H]^-^ | Y=144775*X-19305 | 0.9992 | 0.677-43.3 |
| 4 | | Oroxylin A-7-O-glucuronide | 8.13 | 459.0927 [M-H]^-^ | Y=257003*X-6235 | 0.9978 | 0.135-8.63 |
| 5 | | Wogonoside | 8.52 | 459.0927 [M-H]^-^ | Y=244667*X+1566 | 0.9990 | 0.305-19.5 |
| 6 | | Baicalein | 10.2 | 269.0450 [M-H]^-^ | Y=531463*X-72580 | 0.9927 | 0.140-8.96 |
| 7 | | Wogomn | 12.2 | 283.0606  [M-H]^-^ | Y=722076*X-43697 | 0.9957 | 0.0706-4.52 |
| 8 | | Oroxylin A | 12.8 | 283.0606  [M-H]^-^ | Y=452467*X-65404 | 0.9950 | 0.0630-4.03 |
