# Supplementary material for: Qingrehuoxue formula enhances anti-PD-1 immunotherapy in NSCLC by remodeling the tumor immune microenvironment via TREM2 signaling
Source: BMC Complement Med Ther. 2025 Jul 16;25:270. doi: 10.1186/s12906-025-05020-8 (PMC12269164; doi:10.1186/s12906-025-05020-8)
Supplement: Supplementary file 4 — Supplementary Material 4 [file 12906_2025_5020_MOESM4_ESM.docx]

**Supplementary table 4.** **Content of active substances in QRHXF.**

| No. | Compound Name | Concentration (mg/g) |
| --- | --- | --- |
| 1 | Albiflorin | 1.56±0.00 |
| 2 | Paeoniflorin | 11.8±0.00 |
| 3 | Baicalin | 36.2±0.2 |
| 4 | Oroxylin A-7-O-glucuronide | 2.49±0.03 |
| 5 | Wogonoside | 12.0±0.1 |
| 6 | Baicalein | 6.36±0.05 |
| 7 | Wogomn | 1.86±0.02 |
| 8 | Oroxylin A | 0.606±0.007 |
