# Supplementary material for: Qingrehuoxue formula enhances anti-PD-1 immunotherapy in NSCLC by remodeling the tumor immune microenvironment via TREM2 signaling
Source: BMC Complement Med Ther. 2025 Jul 16;25:270. doi: 10.1186/s12906-025-05020-8 (PMC12269164; doi:10.1186/s12906-025-05020-8)
Supplement: Supplementary file 5 — Supplementary Material 5 [file 12906_2025_5020_MOESM5_ESM.pdf]

1.AKT (Original Image for Fig 7)

	Repeat 1 (used in the manuscript)	Repeat 2	Repeat 3
AKT (56kDa) - Sample	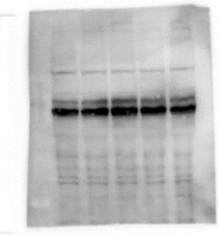	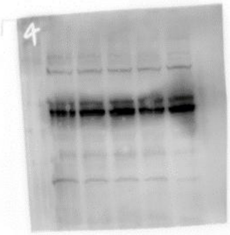	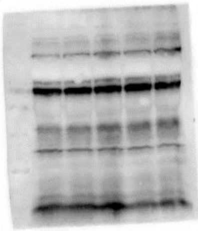
Merge	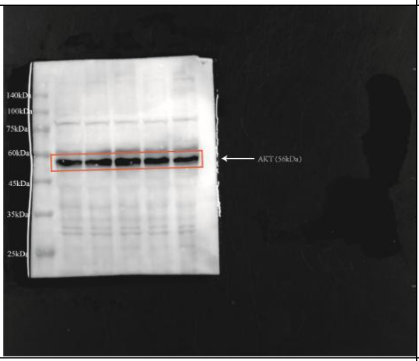	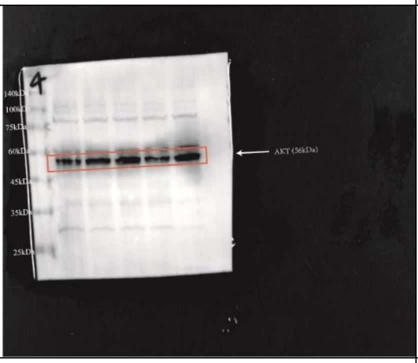	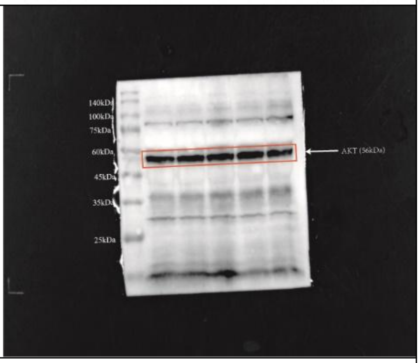
β-actin (46kDa) - Sample	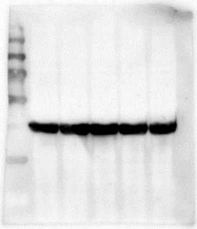	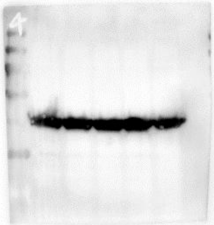	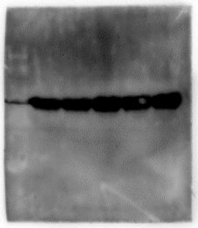
Merge	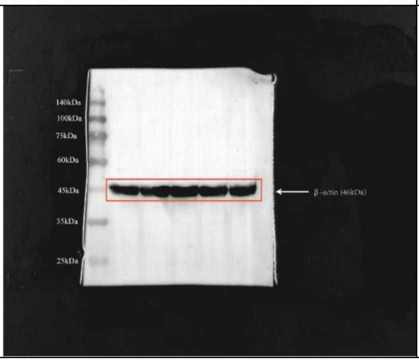	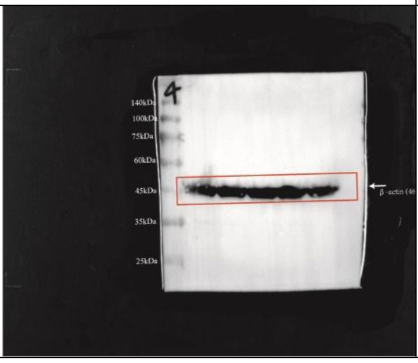	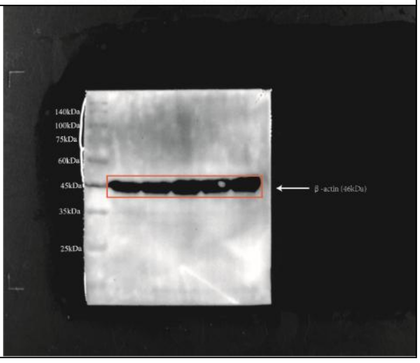

2.Arg-1 (Original Image for Fig 5)

	Repeat 1 (used in the manuscript)	Repeat 2	Repeat 3
**Arg-1 (40kDa) - Sample**	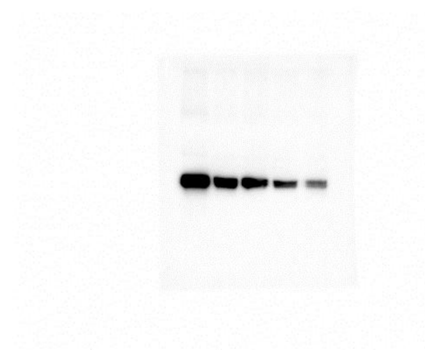	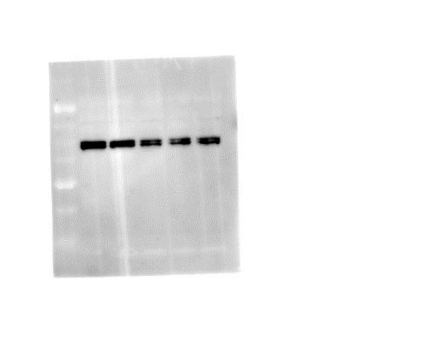	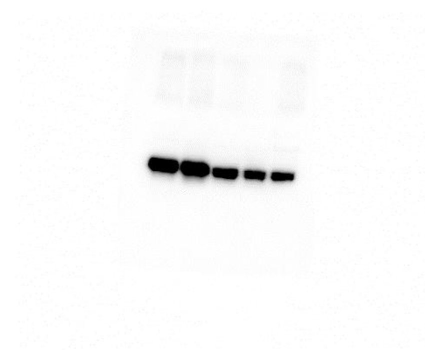
**Merge**	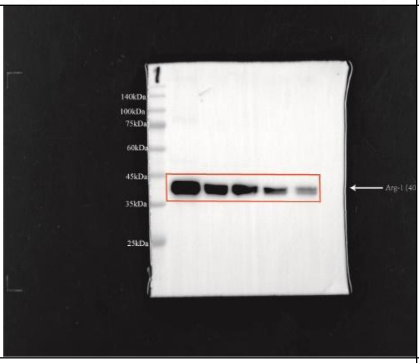	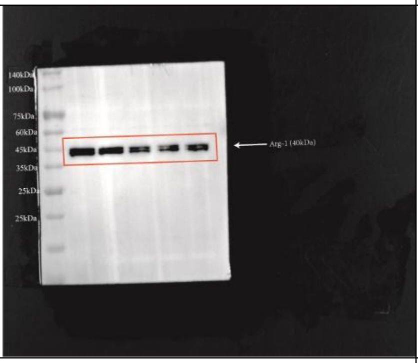	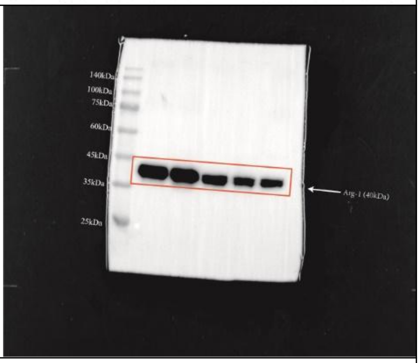
**β-actin (46kDa) - Sample**	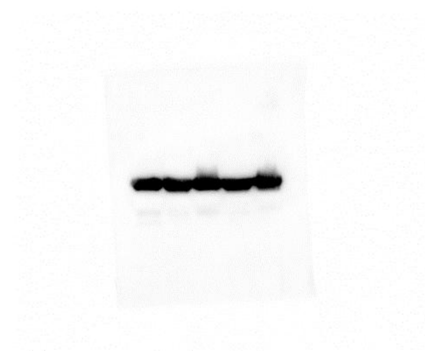	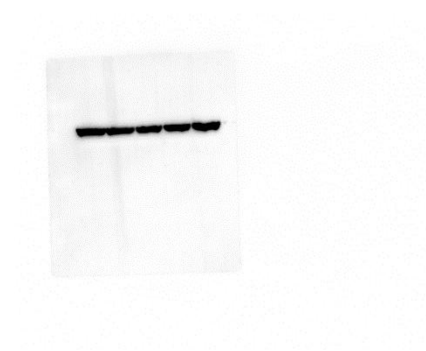	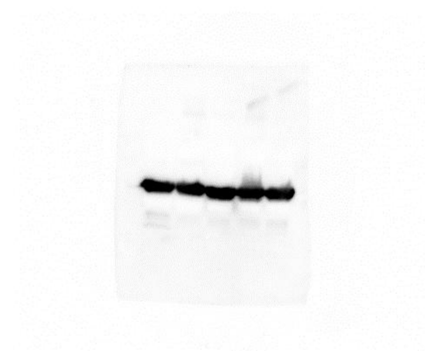
**Merge**	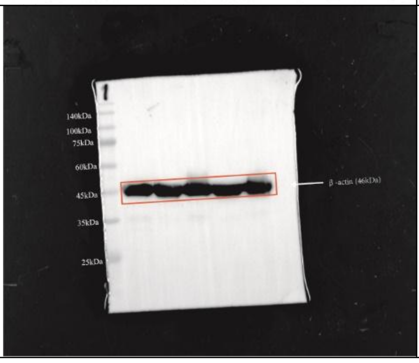	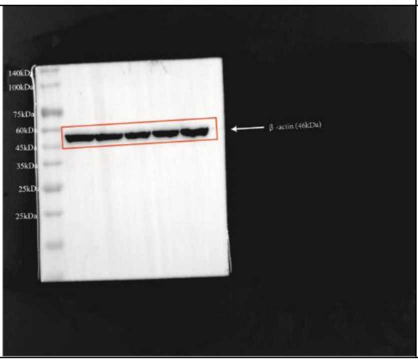	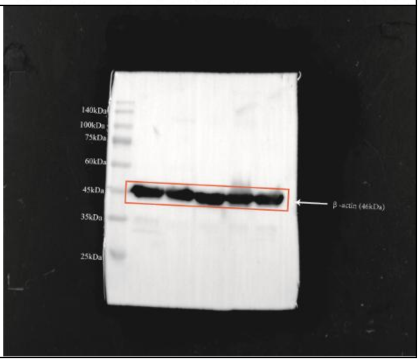

3.iNOS (Original Image for Fig 5)

	Repeat 1 (used in the manuscript)	Repeat 2	Repeat 3
**iNOS (110kDa)-Sample**	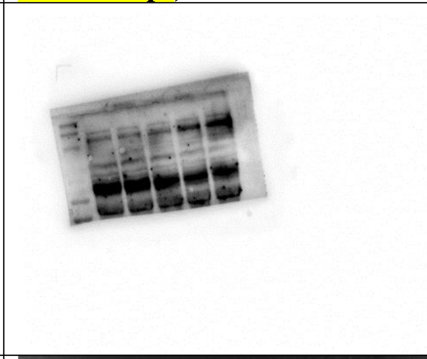	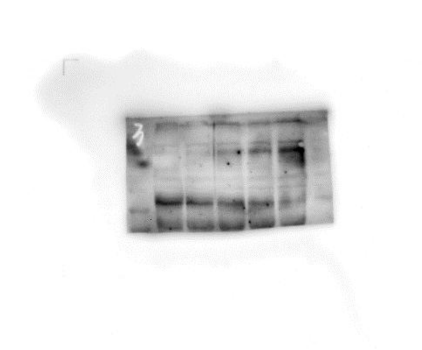	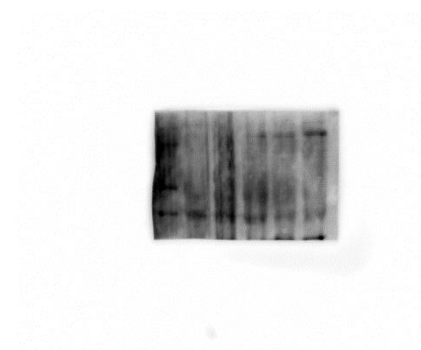
**Merge**	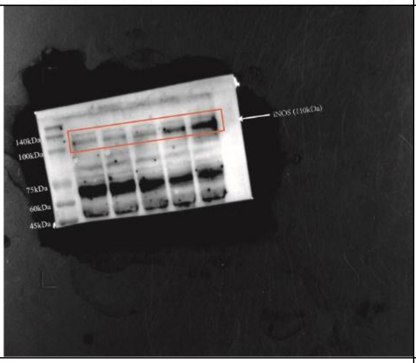	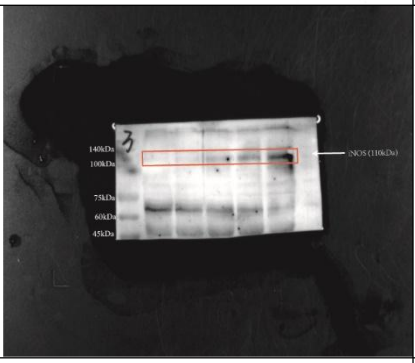	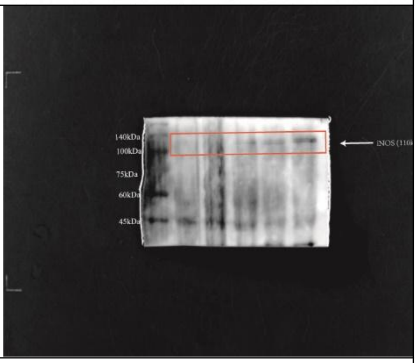
**β-actin (46kDa) -Sample**	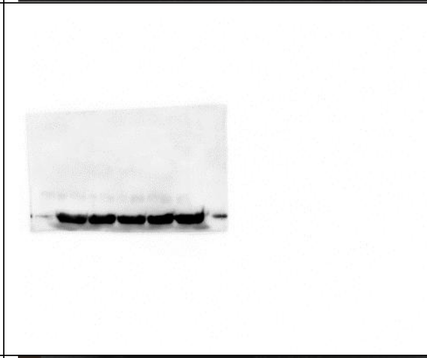	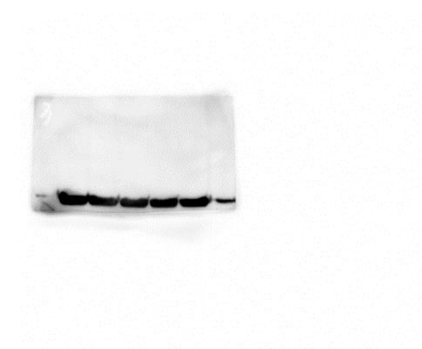	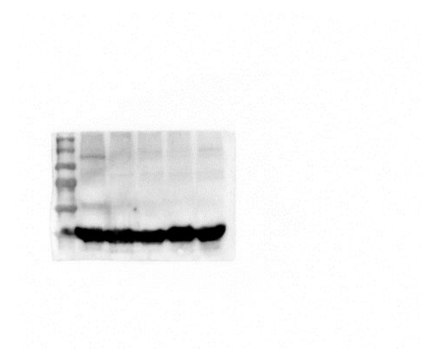
**Merge**	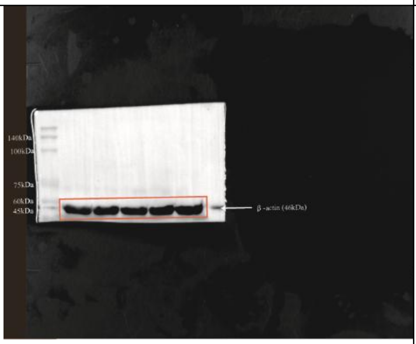	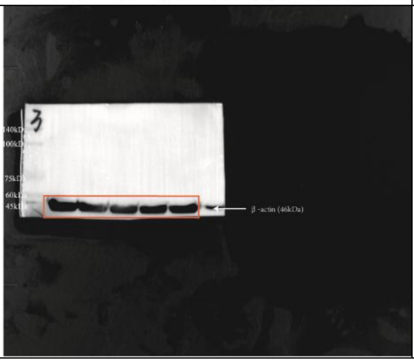	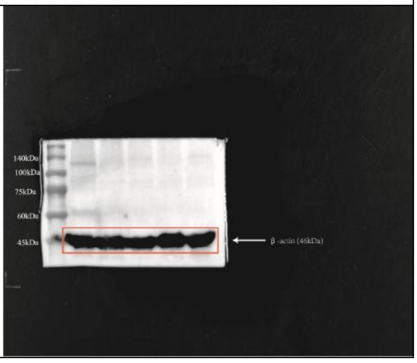

4.MMP2 (Original Image for Fig 3 MMP2)

	Repeat 1 (used in the manuscript)	Repeat 2	Repeat 3
MMP2 (72kDa) - Sample	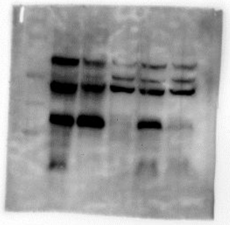	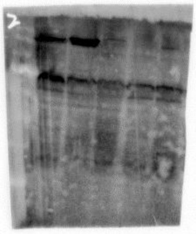	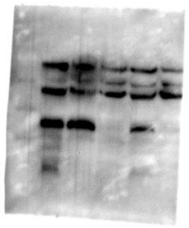
Merge	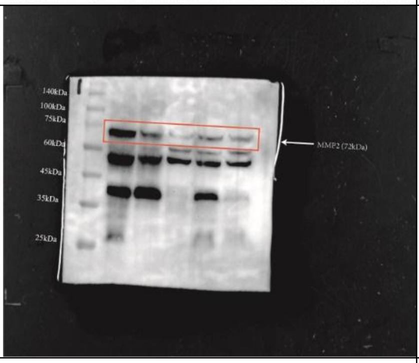	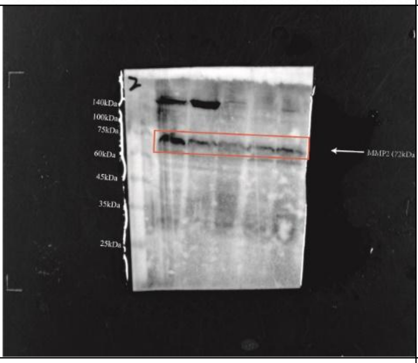	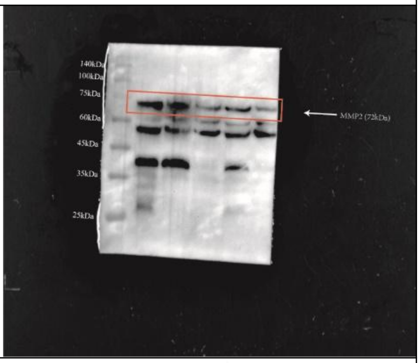
β-actin (46kDa) - Sample	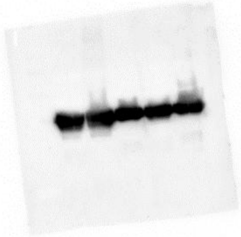	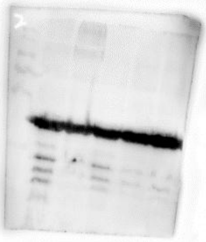	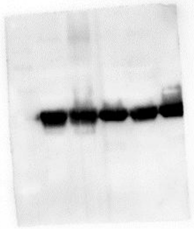
Merge	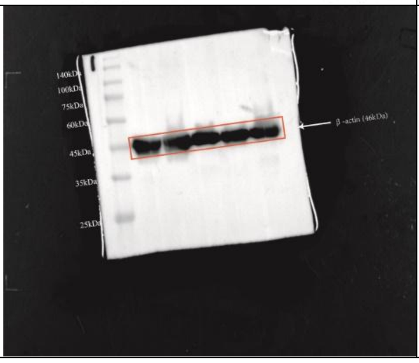	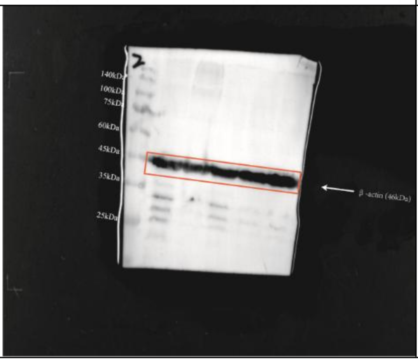	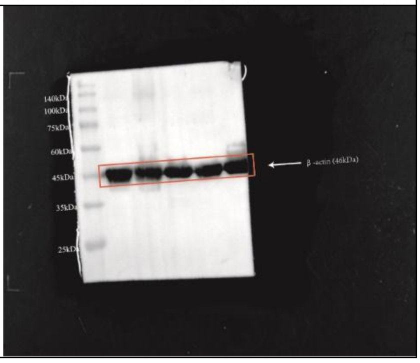

5.MMP9 (Original Image for Fig 3 MMP9)

	Repeat 1 (used in the manuscript)	Repeat 2	Repeat 3
**MMP9 (56kDa) - Sample**	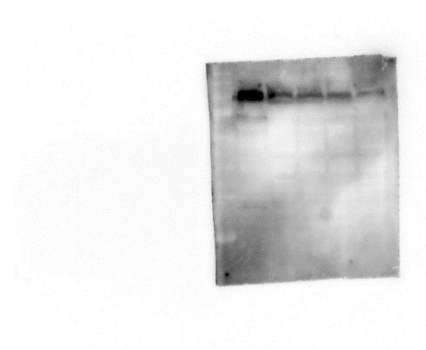	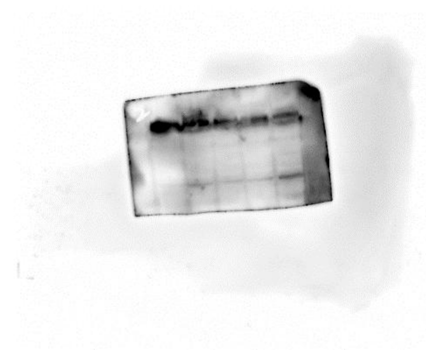	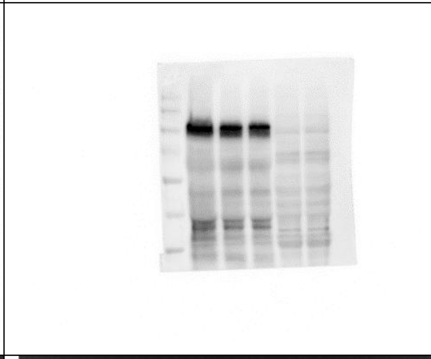
**Merge**	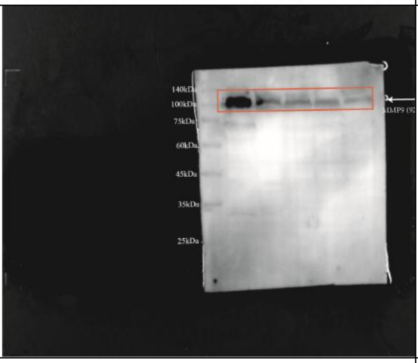	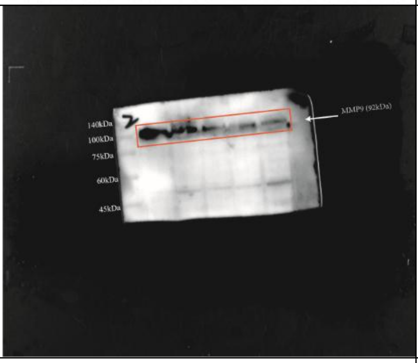	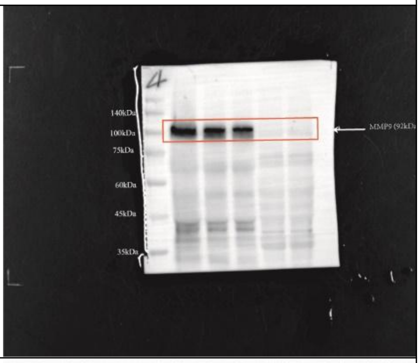
**β-actin (46kDa) - Sample**	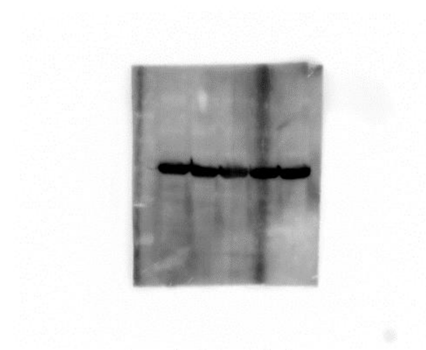	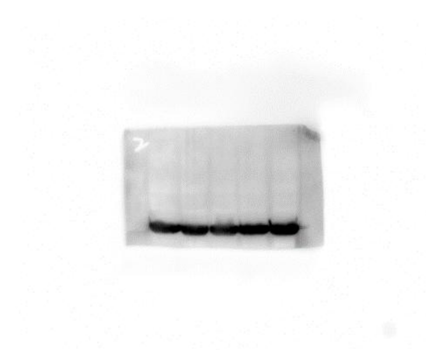	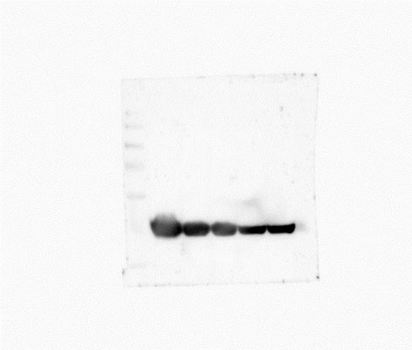
**Merge**	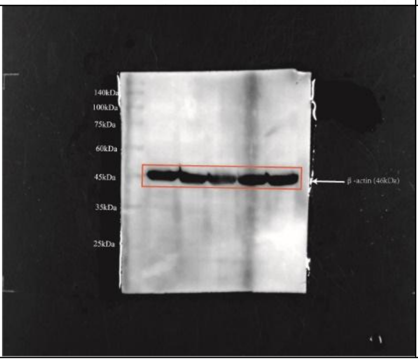	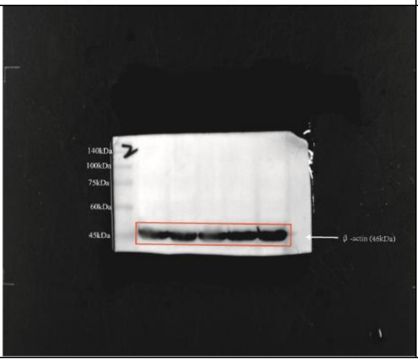	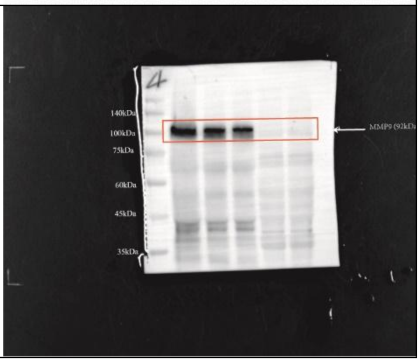

6.p-AKT (Original Image for Fig 7 p-AKT)

	Repeat 1 (used in the manuscript)	Repeat 2	Repeat 3
p-AKT (56kDa) - Sample	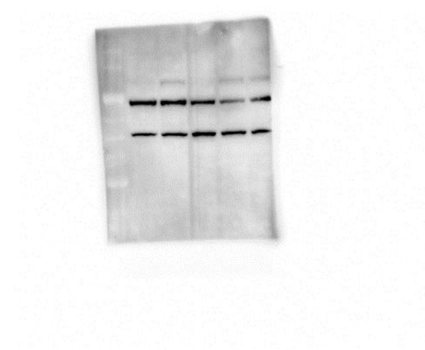	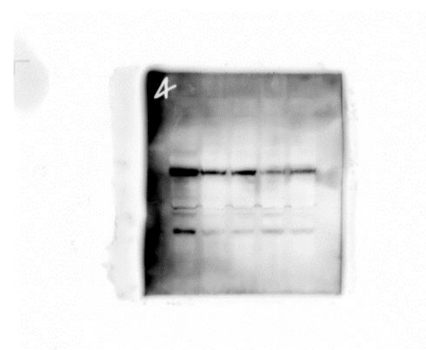	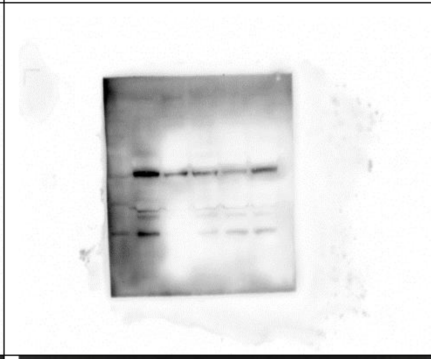
Merge	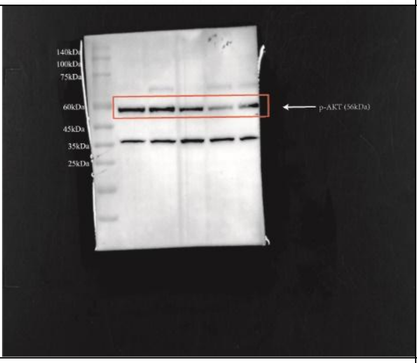	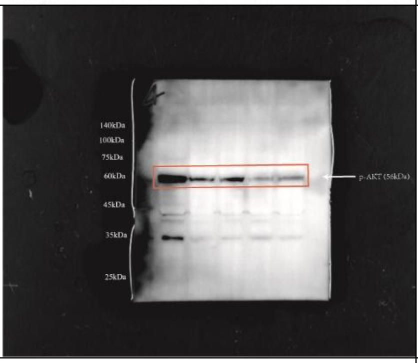	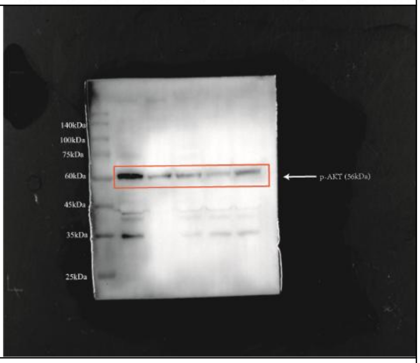
β-actin (46kDa) - Sample	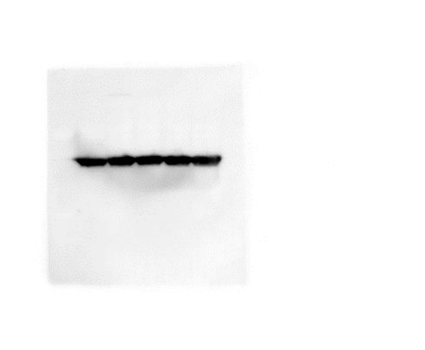	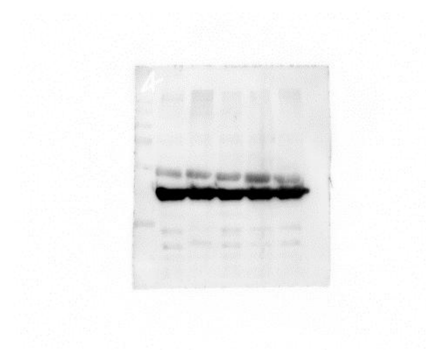	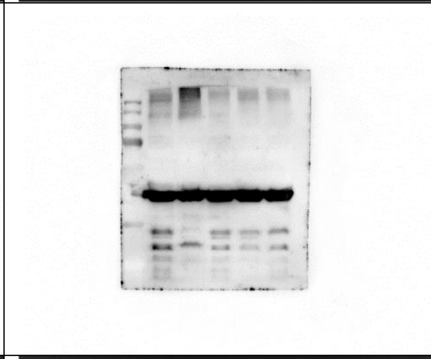
Merge	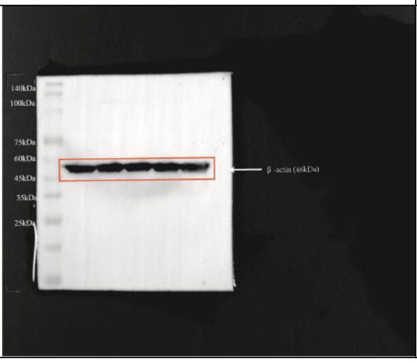	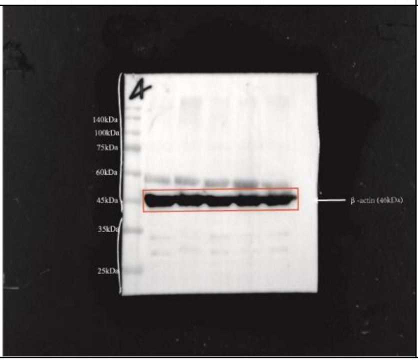	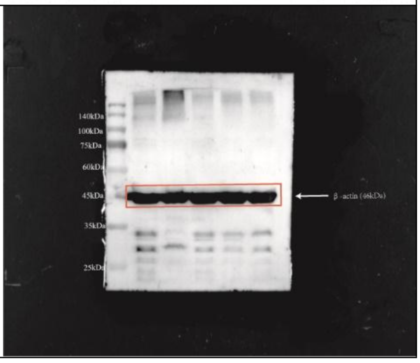

7.PI3K (Original Image for Fig 7 PI3K)

	Repeat 1 (used in the manuscript)	Repeat 2	Repeat 3
PI3K (110kDa)-Sample	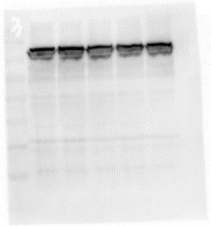	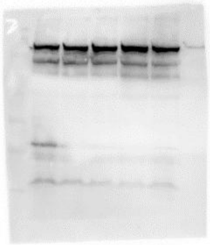	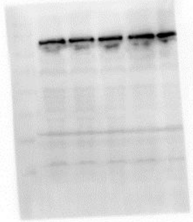
Merge	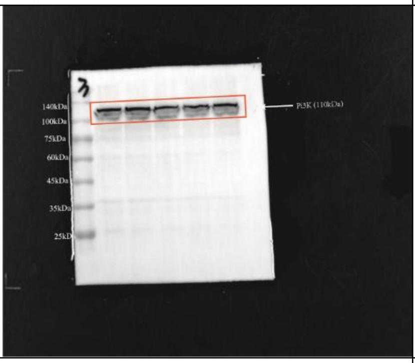	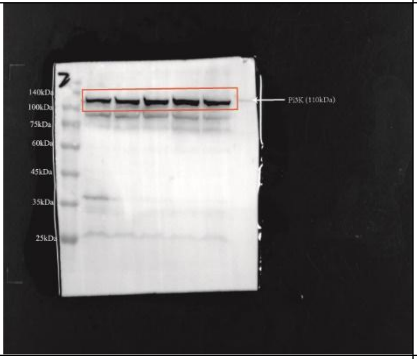	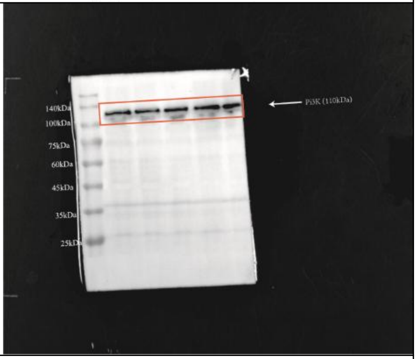
β-actin (46kDa) -Sample	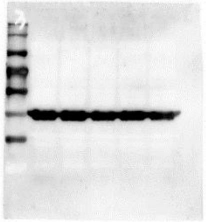	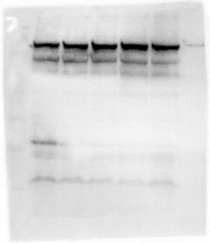	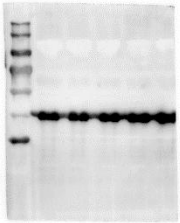
Merge	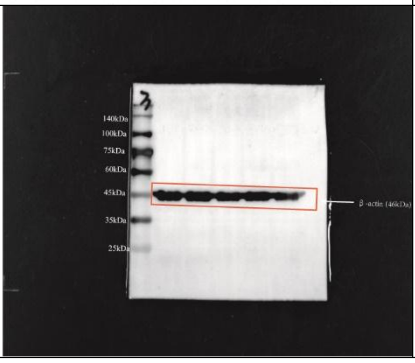	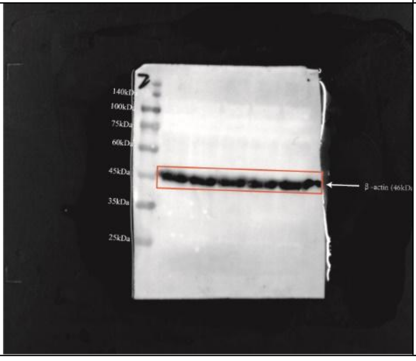	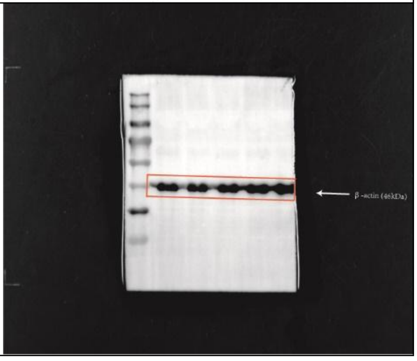

8.p-Pi3K (Original Image for Fig 7 p-Pi3K)

	Repeat 1 (used in the manuscript)	Repeat 2	Repeat 3
p-Pi3K (110kDa)-Sample	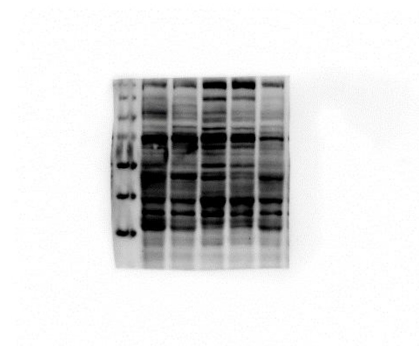	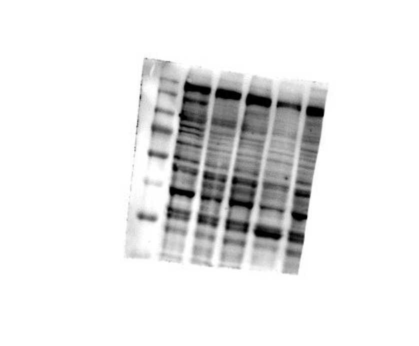	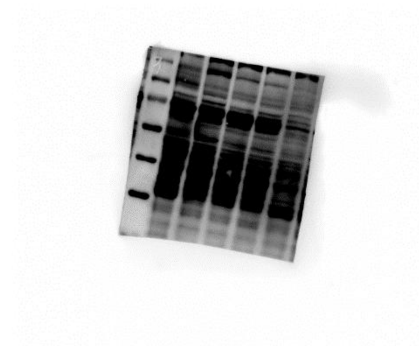
Merge	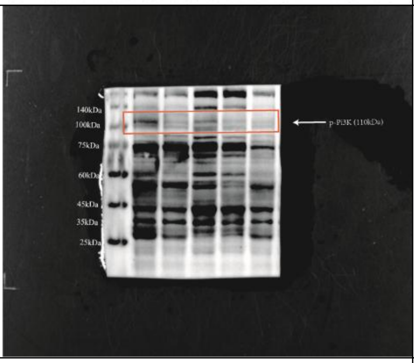	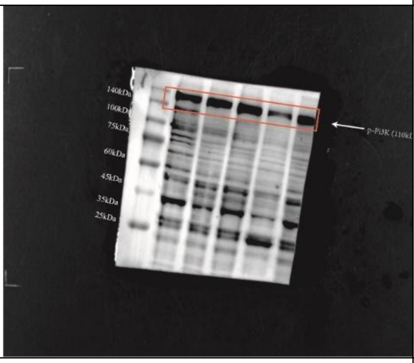	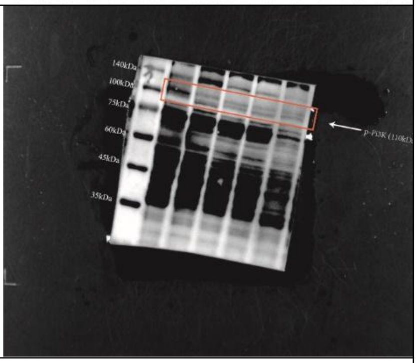
β-actin (46kDa) -Sample	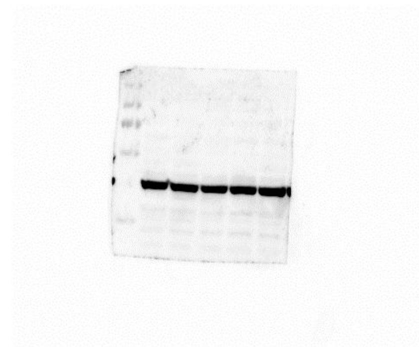	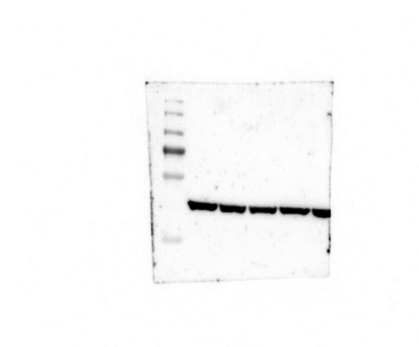	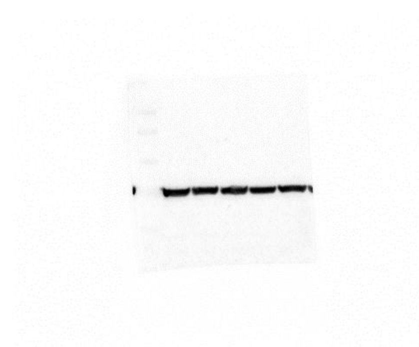
Merge	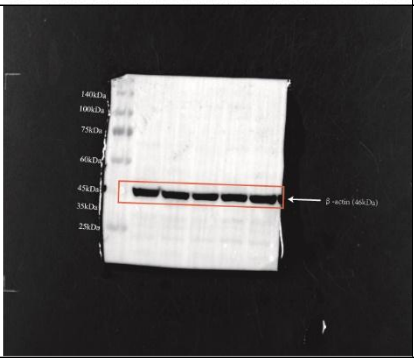	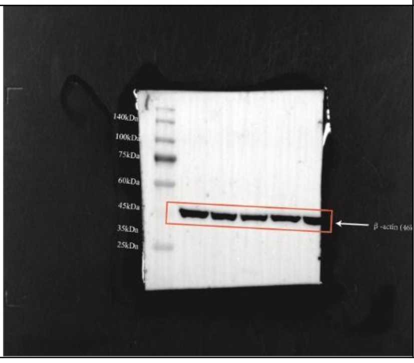	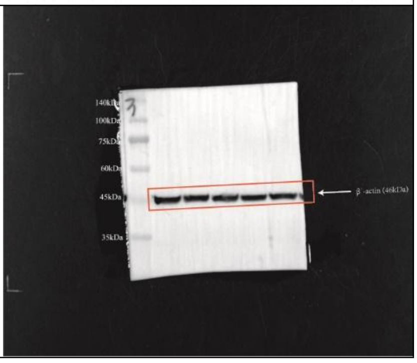

9.p-STAT6 (Original Image for Fig 7 p-STAT6)

	Repeat 1 (used in the manuscript)	Repeat 2	Repeat 3
p-STAT6 (110kDa)-Sample	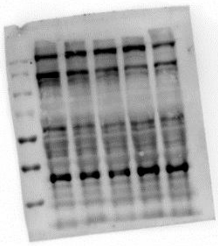	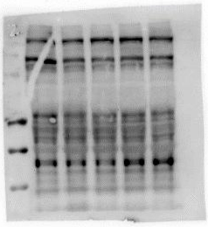	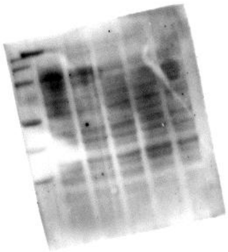
Merge	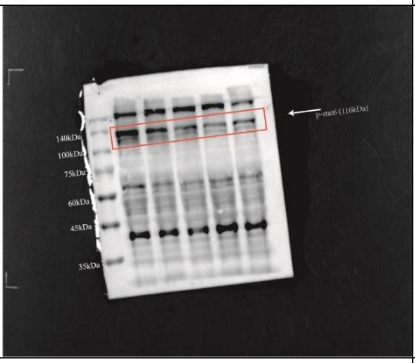	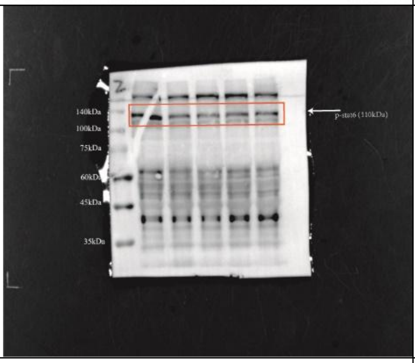	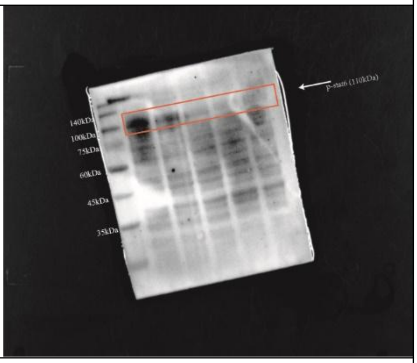
β-actin (46kDa) -Sample	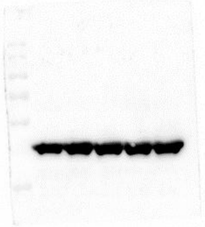	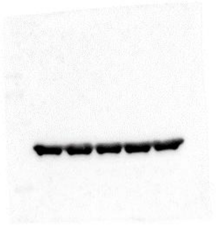	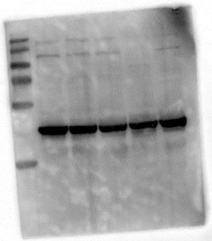
Merge	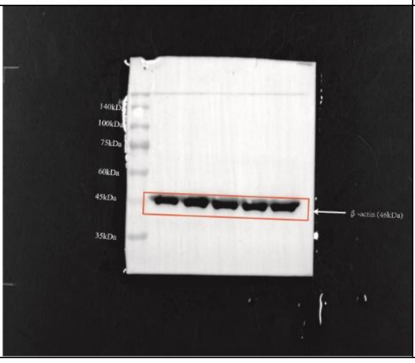	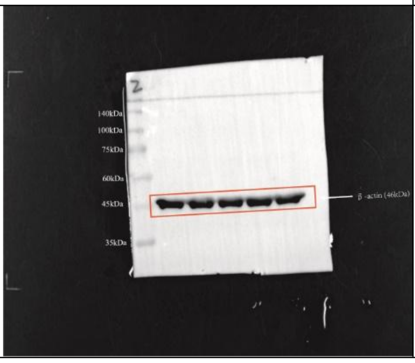	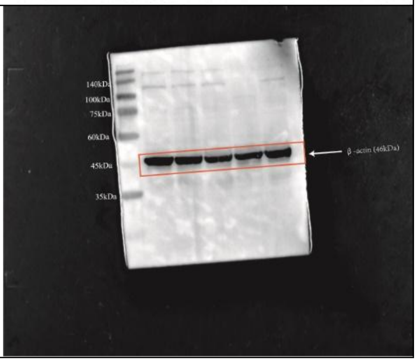

10.STAT6 (Original Image for Fig 7 STAT6)

	Repeat 1 (used in the manuscript)	Repeat 2	Repeat 3
STAT6 (110kDa)-Sample	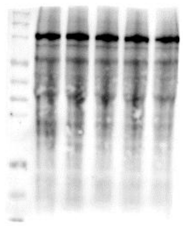	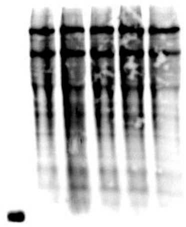	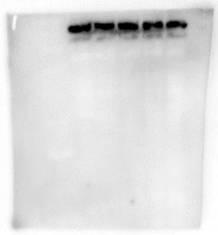
Merge	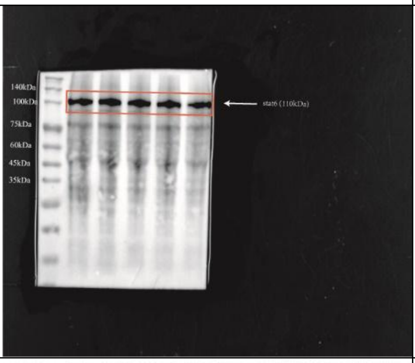	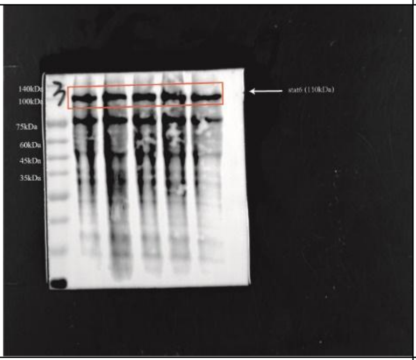	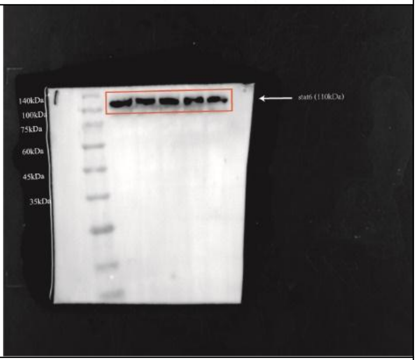
β-actin (46kDa) -Sample	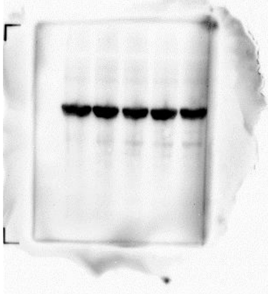	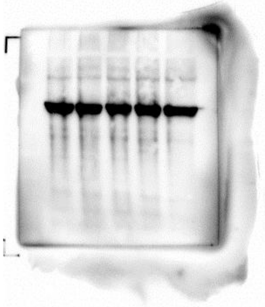	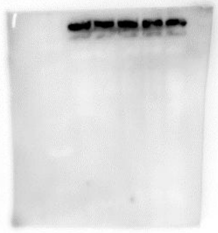
Merge	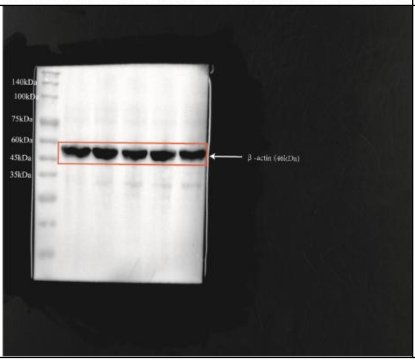	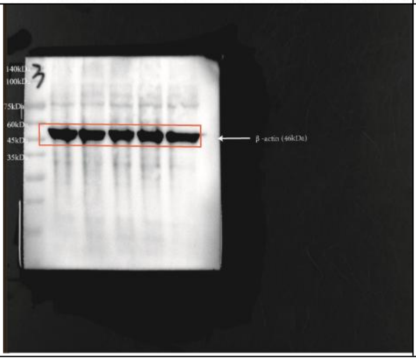	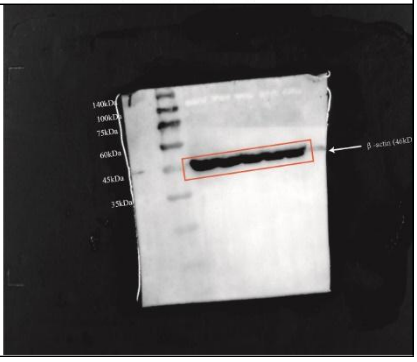

11.TREM2 (Original Image for Fig 7 TREM2)

	Repeat 1 (used in the manuscript)	Repeat 2	Repeat 3
TREM 2 (35kDa) - Sample	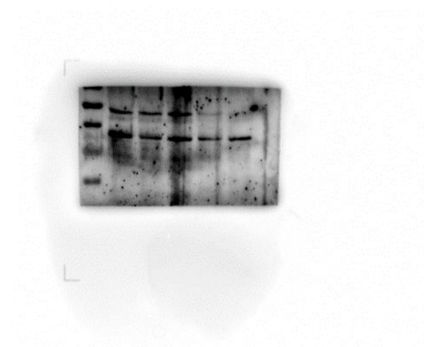	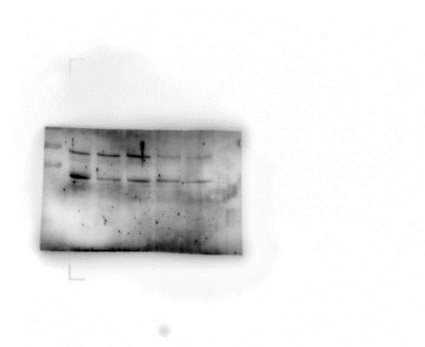	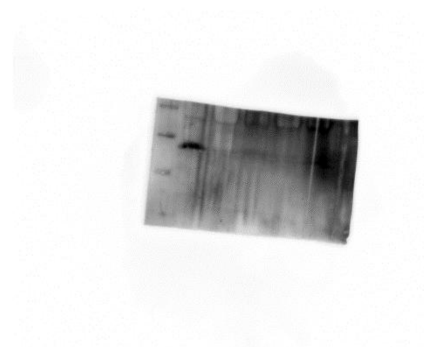
Merge	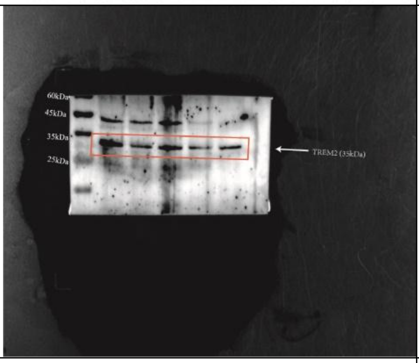	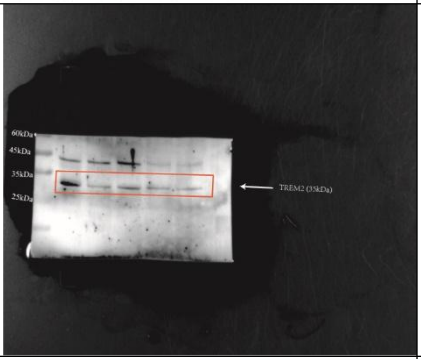	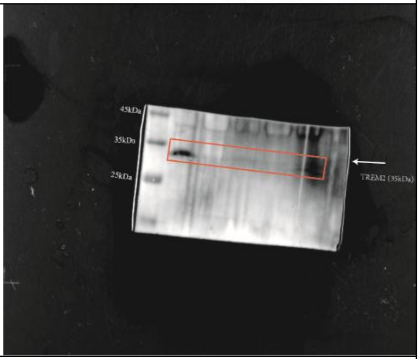
β-actin (46kDa) - Sample	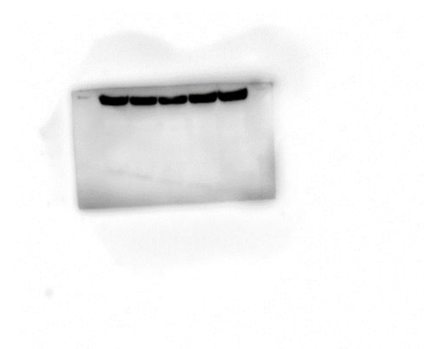	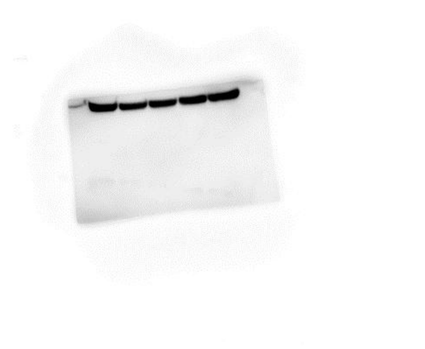	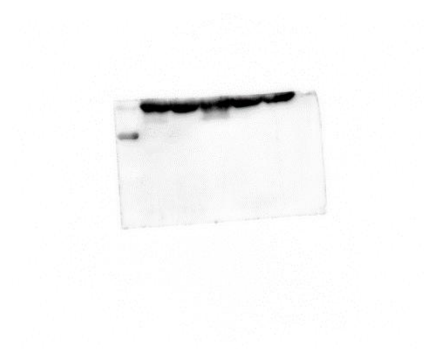
Merge	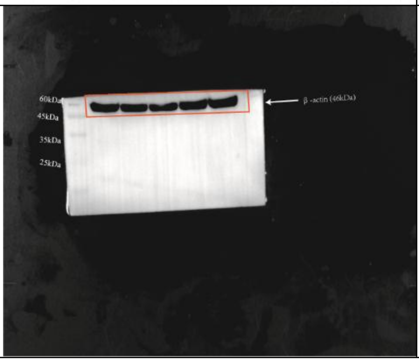	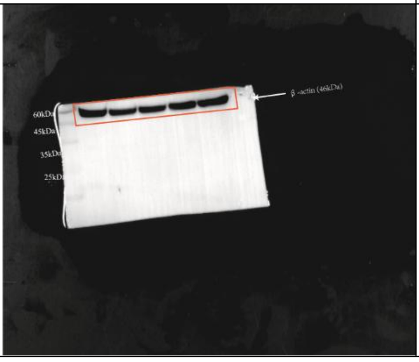	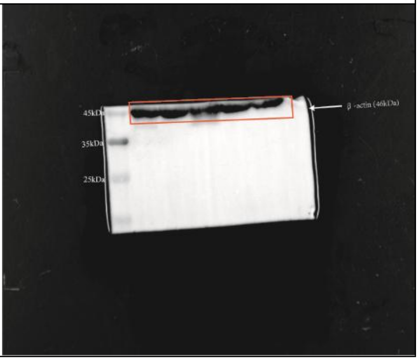
